# Socioeconomic factors predict population changes of large carnivores better than climate change or habitat loss

**DOI:** 10.1038/s41467-022-35665-9

**Published:** 2023-01-24

**Authors:** Thomas F. Johnson, Nick J. B. Isaac, Agustin Paviolo, Manuela González-Suárez

**Affiliations:** 1 Ecology and Evolutionary Biology, School of Biological Sciences, University of Reading, Reading, RG6 6EX UK; 2 UK Centre for Ecology and Hydrology, Wallingford, OX10 8BB UK; 3 Instituto de Biología Subtropical, CONICET-Universidad Nacional de Misiones, Bertoni 85, (N3370AIA), Puerto Iguazú, Misiones Argentina; 4 Asociación Civil Centro de Investigaciones del Bosque Atlántico, Bertoni 85, (N3370AIA), Puerto Iguazú, Misiones Argentina; 5 Present Address: Ecology and Evolutionary Biology, School of Biosciences, University of Sheffield, Sheffield, S10 2TN UK

**Keywords:** Biodiversity, Conservation biology, Socioeconomic scenarios

## Abstract

Land-use and climate change have been linked to changes in wildlife populations, but the role of socioeconomic factors in driving declines, and promoting population recoveries, remains relatively unexplored. Here, we evaluate potential drivers of population changes observed in 50 species of some of the world’s most charismatic and functionally important fauna—large mammalian carnivores. Our results reveal that human socioeconomic development is more associated with carnivore population declines than habitat loss or climate change. Rapid increases in socioeconomic development are linked to sharp population declines, but, importantly, once development slows, carnivore populations have the potential to recover. The context- and threshold-dependent links between human development and wildlife population health are challenges to the achievement of the UN Sustainable development goals.

## Abstract (web summary)

Habitat loss and climate change are widely acknowledged as drivers of wildlife population change, but socioeconomic impacts are relatively unexplored. This study explores drivers of population change in large carnivores and reveals that socioeconomic growth is more associated with population declines than habitat loss and climate change.

## Introduction

Rapid global change is placing wildlife populations under threat^1^. To address this threat, it is important to identify drivers of population trends, revealing sources of declines and opportunities for recovery. However, understanding drivers of population trends (i.e. the direction of abundance change for a given species in a specific location) is challenging, partly because the factors that influence population trends are numerous and hard to measure^2^. For instance, whilst a wealth of ecological knowledge has been amassed with regards to how local-scale environmental change, like changes in land-use^3–5^ and climate^6–8^, influences species population trends. Comparably, we know little about the role of socioeconomic factors and their ability to mitigate or magnify local-scale impacts on wildlife populations. This is problematic as the scarce studies that have focussed on socioeconomics have found large and important effects, where wildlife populations are more likely to increase in areas with strong governance^9^ and areas with a high standard of living^10^.

To effectively detect the signal of environmental change and socioeconomic impacts, it is important to consider the multidimensional context and diversity of potential factors that influence population dynamics. Here, we utilise this multidimensional approach to examine correlative patterns of how land-use change, climate change, governance (of which socioeconomics are a component) and species traits impact population trends of some of the world’s most ecologically and culturally important fauna on the planet—large terrestrial carnivores (e.g. lions, tigers, and wolves). We conduct this assessment in carnivore populations studied in locations across the planet, considering drivers that act over different scales and potentially even interact. We then quantify which of these factors are having the greatest impact on carnivore population change, revealing the important role of socioeconomics in carnivore populations. We then project how socioeconomics may have shaped carnivore abundances across the planet over the past 50 years, finding evidence that rapid socioeconomic development is associated with carnivore declines, but as development slows, carnivore populations may have the capacity to recover—potentially revealing strategies and scenarios that could help bend the biodiversity curve^11^.

## Factors influencing population change

**Fig. 1:**
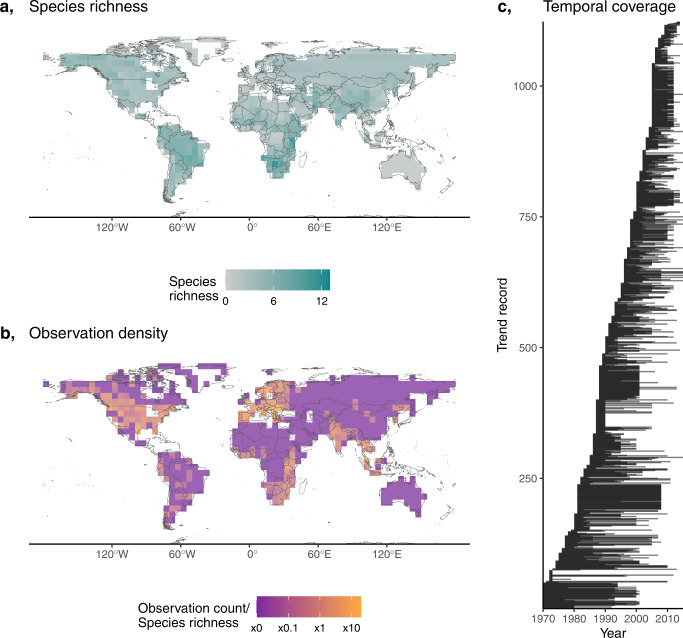
Distribution of carnivore data. **a** Species richness of large carnivores from the families Canidae, Felidae, Hyaenidae and Ursidae of the order Carnivora. Species richness derived by finding the sum of each species (*N* = 87) current IUCN range maps (terrestrial-extant range only, so excluding locations the species is now extinct). For instance, a value of 10 indicates that 10 large carnivore species are suspected to occur within this 5-degree cell according to IUCN range maps. **b** The frequency of collected quantitative and qualitative trends in a given cell divided by the species richness of the cell. Shading is shown on the log-10 scale. Cells with no trends are coloured in deep purple. **c** temporal coverage of quantitative and qualitative observations, where each line represents a trend; the left and right of each line indicates the start and end point of each trend. Source data are provided as a Source Data file.

To determine how land-use change, climate change, and governance influence population trends in large carnivores, we compiled two types of trend data for species (*N* = 87) from the families Canidae, Felidae, Hyaenidae and Ursidae of the order Carnivora: (1) quantitative estimates of change which we converted into annual rates of change (%) in abundance (*N* = 985; representing 50 species), and (2) qualitative descriptions of change: Increase, Stable, and Decrease (*N* = 138; representing 21 species). By including qualitative records, we increased the sample size (Supplementary Fig. 1) and importantly, the taxonomic and spatial representativeness of the data^12^. In total, we compiled trends for 1123 populations, sourced from 7341 abundance estimates available in the CaPTrends^12^ and Living Planet databases^13^. Rates of change were available for 50 of 87 species in our focal group, with locations representing 75 countries, covering all continents with native *Carnivora* species, and variable time periods between 1970–2015 (Fig. 1).

**Fig. 2:**
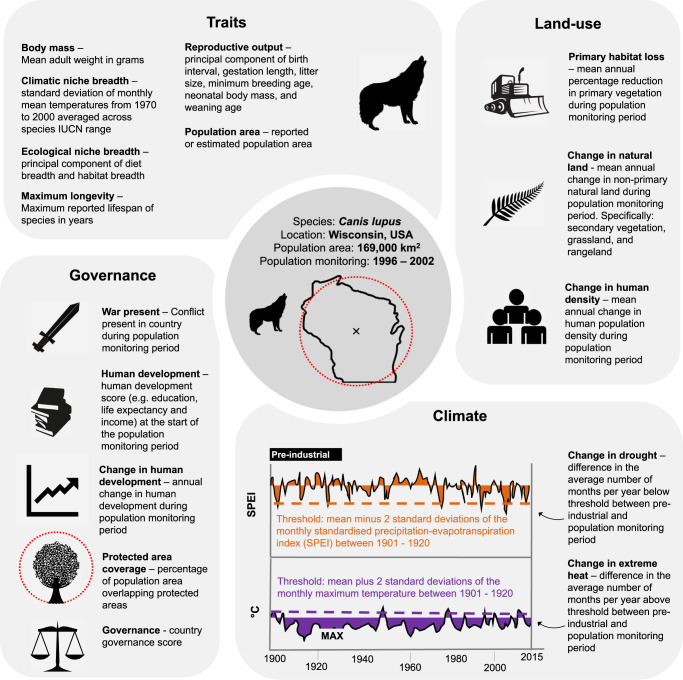
Sixteen covariates with a proposed effect on carnivore population trends. Covariates are highlighted in bold and fall in four groups: Traits, Land-use, Climate, and Governance. Text alongside covariates briefly explains how the variable was derived, whilst full explanation and justifications for inclusion are available in Supplementary methods: Covariates.

For each trend, we extracted covariates describing land-use, climate, and governance (including socioeconomics) features, each of which could influence population trends (Fig. 2). Using the trend data (response) and associated covariates (linked to each trend in space and time when relevant—see Methods: Covariates), we developed a hierarchical Bayesian linear model (see Materials and methods). To allow the integration of both types of trend data within one model, which reduced taxonomic and spatial bias, we included a novel censored response-term to treat the qualitative descriptions as partially known quantitative trends. Models included sixteen covariates, as well as seven interactions specifying how the impacts of environmental change could depend on species traits^14^, the quality of national-level governance^15^, and interactions between different types of environmental change. For example, specialist species are likely to experience greater declines under climate change because specialists are less able to adapt to novel environmental conditions^16^. Given the high number of covariates and interactions (23 in total), we used a variable selection approach to limit model overparameterization.

### Land-use

**Fig. 3:**
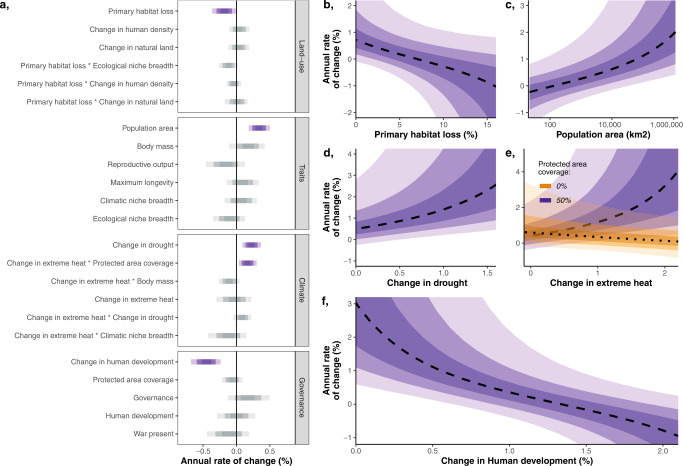
Multiple drivers of population change in large carnivores. **a** Annual rate of change coefficients from fixed effect parameters in a hierarchical Bayesian linear model, with 50%, 80%, and 95% credible intervals. Coefficients with an effect at the 95% credible interval are coloured in purple. Parameters are ordered by effect size within respective facets. **b**–**f** Marginal effects for a selection of important covariates against median predicted annual rate of change: mean annual primary habitat loss (**b**); area of population buffer zone on the log_10_ scale (**c**); mean number of months per year where the average degree of drought in the population monitoring period (the period of time abundances have been assessed for each trend) exceeded the mean plus two standard deviations of the average drought during the baseline pre-industrial period (1901–1920) (**d**); change in temperature (as in change in drought) interacting with protected area coverage (**e**); annual change in human development over the population monitoring period (**f**). All covariates were back scaled from any transformations. Error ribbons represent the 50%, 80%, and 95% credible intervals. Source data are provided as a Source Data file.

As predicted and shown in other taxa^4^, we found that primary habitat loss is associated with declines in carnivores (Fig. 3b), providing further evidence that habitat loss is an important driver of wildlife population declines and biodiversity loss more generally^1^. However, given the previous literature, we expected carnivore declines to be more extreme when habitat was associated with an increase in human density^17^, relatively mitigated when replaced by semi-natural land^17^, and highly dependent on the species ecological niche breadth (its degree of specialization)^18^, but we found no evidence supporting these interactions (Fig. 3a). Our findings suggest that all studied carnivore populations decline in the immediate aftermath of primary habitat loss, regardless of the species ecological specialization and what replaces the primary habitat.

### Climate change

Climate change effects were complex (Fig. 3a). First, an increasing frequency of extreme heat events had no consistent effect on carnivore trends. Instead, we found that extreme heat was associated with declines in populations outside of protected areas but rapid population growth inside, suggesting protected areas mitigate climatic extremes (Fig. 3e). As protected areas are amongst the least impacted fragments of land on the planet, albeit some are heavily degraded^15^, they may naturally offer features that buffer extreme temperatures e.g. micro-climates from canopy cover^19,20^. Increased population densities could also reflect immigration towards these protected areas from less suitable habitat in the short term. The expansion of protected areas could be an effective approach to support future carnivore populations in the face of climate change, and likely could benefit other taxa including birds^21^. However, protected areas cannot promote continuous population growth in the long term, as their area and resources are finite. In fact, our results show that the marginal effect of protected areas (i.e. when all other covariates are held at their average) has no effect on population trends.

Our results reveal another interesting effect of climate change: we expected populations to decline under increasing drought (Supplementary Table 1), but instead found a counterintuitive result where drought frequency was positively associated with population trend (Fig. 3d). There could be mechanisms behind this result, where, for instance, drought could reduce prey fitness^22^, potentially making prey encounter and capture easier for predators, boosting carnivore food availability and resulting in population growth. Such benefits of drought would only be short-term, as predator-prey dynamics would eventually cause carnivore population declines. This explanation is purely speculation though, and more work would be needed to better understand this finding. Our analyses reveal complex relationships between climate variables and habitat protection that should be further investigated in other taxa and monitored over time to detect and respond to changes.

### Governance

Previous analyses have identified a positive association between wildlife abundance trends and human development^10^ and governance^9,23^. However, across the 1123 carnivore populations we studied, we found no effect of governance or human development on carnivore population trends (Fig. 3a), regardless of whether human development was modelled as a linear or quadratic term (Supplementary Table 6)^10^. Instead, we found that rapid human development growth—the improvement in quality of life and economics—was associated with carnivore population declines (Fig. 3f). These declines do not appear to be driven by underlying factors stimulating human development growth like detrimental land-use change, as these factors were directly modelled and accounted for at more relevant spatial scales e.g. we assess primary land loss within the area of the population whilst human development change is described nationally. Instead, we hypothesise that human development change provides a more holistic snapshot of environmental and societal transformation. Potentially capturing changing relationships and tolerances for wildlife, which could influence the prevalence of important drivers of wildlife abundance change like poaching pressure and human-wildlife conflict—features that we know are important^1^ but are largely hidden because they are difficult to quantify locally, nevermind globally. For instance, using Kenya as an example, human development growth has coincided with habitat changes like those we account for in our model^24^, but crucially, human relationships with nature have also weakened^25^. These weakening relationships with nature could potentially synergise with habitat loss, where species are not only having to persist in a changing habitat, but tolerance for these species could also drop, potentially leading to persecution or human-wildlife conflicts^26^.

### Relative contributions

To assess the contribution of important covariates, we developed three counterfactual scenarios to describe how the absence of specific features (or threats) would alter the 1123 observed trends holding all other features constant. Scenarios included: (1) No loss in primary habitat—primary habitat loss set to zero; (2) No climate change—both change in frequency of extreme heat and drought set to zero; and (3) No growth in human development—change in human development set to zero. We subtracted these counterfactual predictions from the observed trends to define ‘Difference in annual rate of change (%)’, whereby a positive value indicates carnivore populations would be in better shape (fewer and smaller declines) had there been no changes in habitat, climate and human development, whilst a negative value indicates observed changes benefitted carnivore populations (more declines under no change).

**Fig. 4:**
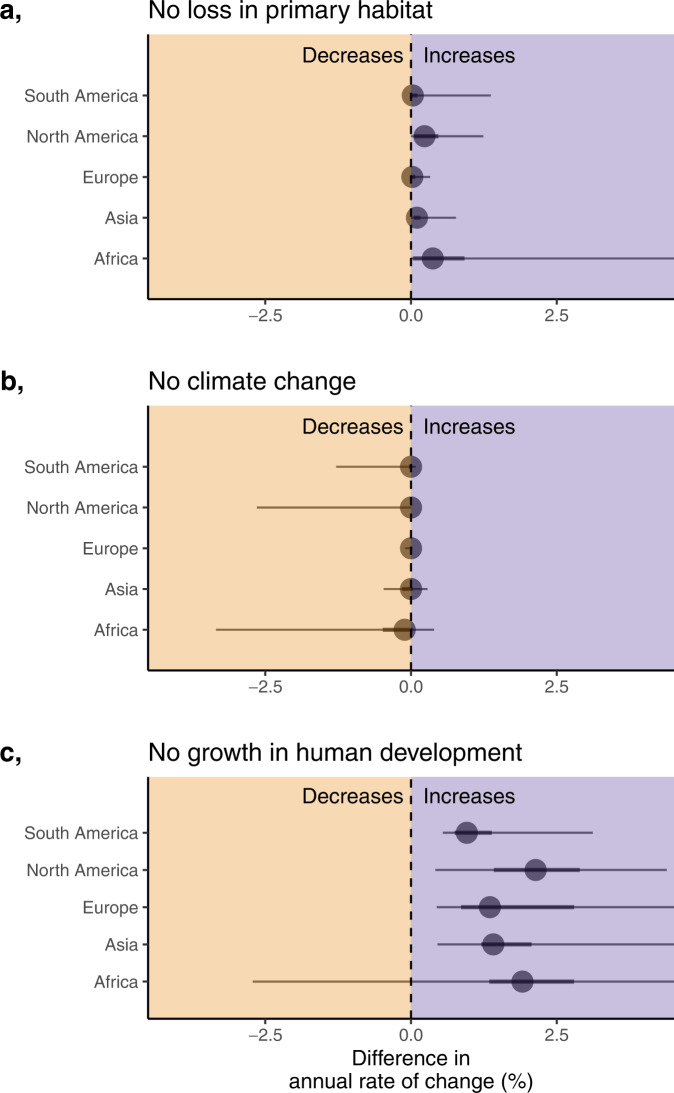
Human development is the primary driver of population change in large carnivores. Counterfactual scenarios describing the difference in the annual rate of change (%) across the 1123 studied populations had there been no primary habitat loss (**a**), climate change (**b**), or growth in human development (**c**). Points falling on the right of the dotted line indicate that the population would be predicted to increase had observed habitat loss, climate change, and human development growth not occurred, and populations on the left of the line would be predicted to decrease. Points represent the median difference in the annual rate of change (predicted trend using counterfactual data minus the predicted trend using the observed data), with 50 and 95% quantiles. Source data are provided as a Source Data file.

The counterfactual scenarios reveal the great importance of human development: its contribution to population changes dwarfed those of habitat and climate change (Fig. 4). No consistent increases or decreases in population trends resulted from assuming observed habitat loss and climate change had not occurred. As an exception, African carnivore populations would have benefitted overall from preservation of habitat. The lack of a sizable habitat loss impact in other regions, despite a large effect size (Fig. 3b), is a consequence of most populations having experienced very little habitat loss—only 6% of populations experienced high habitat loss (−5% or lower). Similar rationale explains the generally high skewness within the habitat and climate counterfactuals, where populations changed under the pressure of high habitat and climate impacts, but most populations did not experience this pressure. In contrast, human development changes had clear and consistent impacts across carnivore populations in all continents, with nearly all counterfactual trends being higher. The low prevalence of habitat and climate impacts may suggest populations are not experiencing extreme habitat loss and climate change—but this is unlikely given the rates of global change^27^. A more likely explanation is that we simply lack data on populations experiencing these extreme conditions, which could mean we are underestimating the effects of habitat loss and climate change, as well as their prevalence.

## Socioeconomic development and non-linearity in carnivore trends

**Fig. 5:**
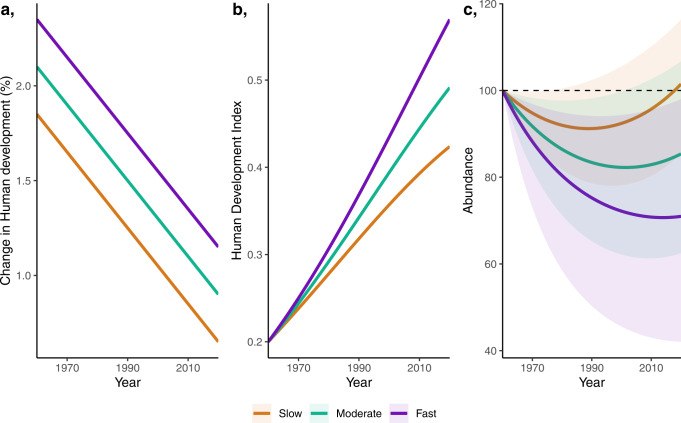
Projections of carnivore abundance vary with rate of change in human development. **a** Instantaneous Change in Human development (as in the covariate and parameter in Fig. 3) over time under three pathways: Slow, Moderate and Fast. A key feature of the human development data is a deceleration in the human development growth rate as human development increases (**a** and Supplementary Fig. 12a). **b** Projections of the Human development index (as in the covariate and parameter in Fig. 3) from a baseline of 0.2, derived using the three pathways of instantaneous change in human development (**a**). **c** Potential impact of different development strategies on carnivore abundances, relative to an arbitrary baseline abundance of 100 (dashed line). Solid line describes the median abundances, shading represents the 95% credible intervals in abundance, based around the uncertainty in the human development coefficient. Source data are provided as a Source Data file.

Our analyses reveal that fast human development growth is associated with declines in carnivores, but notably, once human development growth drops below c1.2%, carnivore populations can increase (Fig. 3f). We derived this result by assessing how patterns of human development change may influence carnivore population trends across countries (i.e. a space-for-time substitution). However, metrics of human development not only differ across countries, but are also dynamic within countries, with human development growth tending to decelerate over time. We explored the consequences for carnivore populations of decelerating within-country human development trajectories under three idealised socioeconomic pathways. Our pathways (time-series) describe the Change in Human development over time, with three options: Slow, Moderate and Fast. These pathways solely differ in their mean rate of change in human development (i.e. *pace*); we specified that human development change should *decelerate* equally in each pathway (Fig. 5a; see Methods for justifications). We project the pathways from an arbitrary baseline human development of 0.2 in the year 1960 (Fig. 5b), broadly resembling the observed human development pathways for a selection of countries (Supplementary Fig. 12). We then use the Change in human development parameter from our fitted model (median standardised effect size: −0.44; Fig. 3f) to project carnivore abundances up to 2020, from an arbitrary baseline abundance of 100 in 1960 (Fig. 5c). We hold all covariate parameters constant over time and equal to the average global value i.e. isolating the marginal effect of Change in human development. These projections are not representative of actual abundances and solely represent a hypothetical scenario.

Our simulations show that apparently small differences in human development change could have large impacts on carnivore population trends (Fig. 5). Firstly, regarding pace, our pathways with a faster mean rate of change in human development led to greater declines in carnivore abundance, and only under a ‘Slow’ pathway are populations projected to increase above the initial baseline (Fig. 5c). Secondly, and perhaps encouragingly, a key feature of the human development data is that the rate of change decelerates as human development increases (Supplementary Fig. 12a). This decelerating change in human development, which was equal across all three of our pathways, creates non-linearity in carnivore abundances, reversing carnivore trends from steep declines to stability, and even towards recovery (Fig. 5c). These turning points (the point a trend flips from decline to recovery) happen when the Change in human development drops below c1.2%—occuring earlier and after smaller declines with ‘Slow’ Change in Human development. Under fast and prolonged growth in human development (a more extreme scenario than any we present here), there is a risk that carnivores could decline to local extinction (abundance at zero) before human development growth slows and carnivore populations are able to recover.

Our simulations offer insight into the potential role of development pathways on abundance change. Our results broadly resemble a Kuznets curve: as economies become industrialised, the level of environmental degradation first increases, then reaches a plateau, and then begins to decrease^28^. In our simulations, fast development growth led to carnivore population declines, but then abundances begin to recover as development rises and growth slows, resembling the outcome of the Kuznets curve, albeit not the same mechanism (our curve is based on development change, not development level). The evidence of a biodiversity specific Kuznets curve is mixed, with some research suggesting strong support^29^, whilst others find mixed^30^ or no clear evidence^31^. More work is needed to validate and characterise the biodiversity Kuznets curve, which offers a framework to understand non-linearity and opportunities for recovery in biodiversity trends. Invoking the Kuznets curve analogy from our results depends on the notion that trends of carnivores in developed countries provide a basis for predicting the future for developing countries (i.e. the space-for-time substitution). An important caveat is that developed countries had already lost some large carnivore biota. Available data in these countries is from species that were extant in 1960, which are likely to be more resilient to anthropogenic pressures than extinct species. The data for countries with high development scores may be biased, making them a poor model for predicting the trends for countries that are going through the development transition.

Our projections also highlight the potential challenges facing countries currently experiencing rapid development growth: they face a trade-off between improving the living standards of people and maintaining carnivore populations (Fig. 5c). This trade-off could hinder the achievement of the UN sustainable development goals (SDG) in rapidly developing countries. For example, improvements in health, education and income (SDGs 1–5) could negatively impact large carnivores (and possibly biodiversity as a whole), hindering progress towards SDG 15. Work is needed to establish and mitigate the mechanisms by which human development growth (a proxy of socioeconomics more generally) can induce carnivore declines, helping to resolve conflicting SDGs, whilst continuing to promote human development growth.

We analyse population trends for large mammalian carnivores showing that abundance is likely to decline under primary habitat loss, and that climate change has complex effects depending on the degree of protected area coverage. For climate change, we expect many of the threats are yet to be fully realised and could become stronger in the future^8^. These effects may not be very surprising but the particularly strong importance of human population characteristics (i.e., human development) was unexpected. Socioeconomic factors seem to be the theatre where effects of climate and land-use change are played out. We must consider the wider socioeconomic scope when evaluating wildlife population change, and biodiversity more generally. Whether our socioeconomic effects hold true for taxa other than large carnivores is unknown and should be tested. It is possible that socioeconomic effects are amplified in large carnivores as they are particularly likely to experience socioeconomic related threats like hunting and human-wildlife conflict. Regardless, focusing solely on stressors like land-use and climate change may be effective at identifying causes of declines, but provides few opportunities to identify the features that support recovery, and these features may hold the key to bending the biodiversity curve. In large carnivores, recoveries are already underway in parts of the world^23^, but challenges remain. Capturing the multitude of complex features influencing wildlife populations—including environmental, societal and cultural changes—will be essential to understanding and addressing these challenges.

## Methods

Our study is focussed on population trends of large carnivores; a culturally important group^32^, essential for regulating ecosystem function^33^. Large carnivores represent an important study group as their population status is unclear, with reports of devastating declines^33^ contrasted with remarkable recoveries^23^. Further, as a well-studied taxa with abundant trend and trait datasets, large carnivores present a good system to evaluate important drivers of trends without being impacted by poor inference from missing data^34^. Finally, as large carnivores are considered indicator species of the overall status of biodiversity within an area^35^, our inference may provide insight beyond our focal taxa.

### Population trends

We sourced population (defined by the authors of the original studies, who reported on population trends for one or more studied groups of individuals) trend information for species in the families Canidae, Felidae, Hyaenidae, and Ursidae of the order Carnivora from two large trend datasets: CaPTrends^12^ and the Living Planet Database^13^. The CaPTrends database is the product of a semi-systematic literature search for population trends of large carnivore species (from the families listed above); the dataset possesses trend information for 50 species from locations around the world, and trends are reported in a variety of ways. The Living Planet Database contains population abundance time-series for vertebrates from thousands of sites around the world and is one of the larger population trend datasets. Combined, these datasets produce a cumulative 1123 trends (after removing duplicates and records we deemed unreliable or unsuitable), derived from >10,000 individual population estimates. In the Living Planet Database, and for most records in CaPTrends, trends are reported as a time-series of abundance (or density) estimates. We modelled these time-series with log-linear regressions, where abundance (the response) was log_e_ transformed, and year of abundance estimates was selected as the predictor. We included a continuous Ornstein-Uhlenbeck (OU) autoregressive process to control for temporal autocorrelation in these models. The OU process estimates covariance between abundance values, under the assumption that abundances in time point 1 will be more similar to abundances in time point 2, than time-point 3, 4, 5, etc. Accounting for covariance resolves non-independence within time-series. We extracted the slope coefficient which represents the annual instantaneous rate of change, sometimes called the population growth rate (*r*_*t*_). Alongside the abundance time-series, CaPTrends also has three other quantitative datatypes, all of which we converted into an annual instantaneous rate of change (*r*_*t*_): (1) a mean finite rate of change; (2) estimates of percentage abundance change between two points in time; and (3) time-series’ of population change estimates (e.g. in year 1 the population doubled and in year 2 it halved). We converted all annual instantaneous rates of change into an annual rate of change percentage to improve interpretability. These annual rates of change ranged from −75 to 68%, but the majority of values fell within −10 to 10% (Supplementary Fig. 1a).

Alongside the quantitative records, 138 populations in the CaPTrends dataset were only described qualitatively with categories: Increase, Stable, and Decrease. These qualitative records were more common for populations located in traditionally poorer-sampled countries (e.g. with lower human development), so whilst they are less informative (only describing the direction and not the magnitude), we deem them important to reduce bias (Fig. 1). As a result, we used a combination of percentage annual rates of change (*N* = 985) and qualitative categories (*N* = 138) as our response in our model (see below), representing 50 large carnivore species.

### Covariates

For each population, we extracted sixteen covariates (each z-transformed) that fell into four categories: land-use, climate, governance, and traits. Our covariates were designed to cover a diverse array of factors that could influence population trends in large carnivores (Supplementary Table 1). Each covariate is described briefly in Fig. 2 with full descriptions of how variables were derived in the Supplementary material: Covariates.

One of the challenges in identifying how covariates—which can vary in space and time—impact population trends is matching the spatial and temporal scale of the covariate with the population i.e. how much of the population is affected by the covariate at a given point in time. To tackle the spatial element of this problem, we used data on the area of extent of each population (e.g. how large is the spatial extent of the population or monitoring zone) to generate a circular distribution zone around the population’s coordinate centroid. We refer to this as the ‘population area’ hereafter. We then sampled covariate values within each population area, with more sampling points in larger areas (range: 13–295 sampling points, Supplementary Fig. 2b). For covariates which varied over time, we extracted the covariates across the ‘population monitoring period’, which refers to the period (from start to end year) the population was monitored for. However, as evidence suggests there can be a lag period between impact or change and any detectable changes in population abundance^3^, we tested how 0-, 5-, and 10-year lags in covariates changed model fits and effect sizes. We implemented these lags by extending the start of the population monitoring period backwards for each given lag e.g. for a 10-year lag, a normal population monitoring period of 1990–2000, would then capture covariates between 1980–2000. Sensitivity analysis showed a 10-year lag had the greatest balance of improved model fit, with high taxonomic and spatial coverage (see Supplementary: Sensitivity analysis).

### Modelling

At its core, our model is a linear mixed effects model, regressing annual rates of change against a combined 23 covariates and interactions, using random intercepts to account for phylogenetic and spatial nesting. The model was written in BUGS language and implemented in JAGS 4.3.0^36^ via R 4.0.3^37^. The model structure is summarised below, with a full description in Supplementary: Modelling.

#### Response

We modelled our annual rate of change response with a normal error prior. However, to allow the two different types of population trend data (quantitative rates of change and qualitative descriptions of change) to be included in the same model, we treated the qualitative records as partially known. Specifically, we censored the qualitative records to indicate that the true value is unknown, but it occurs within a specified range, with annual rate changes ranging from −50 to 0%, −5 to 5%, and 0 to 50% within the decrease, stable and increase categories, respectively. The overlapping nature of these thresholds is by design, as we wanted to acknowledge that there is likely a grey area between the different categories. For instance, in one study, a 2% trend could be called stable, whilst a different study would consider this as increasing, our overlapping thresholds address this grey area. Admittedly, our category thresholds were arbitrarily selected—this is as a consequence of there being no strict rules on what population change is needed to be assigned a given category. However, despite being arbitrary, they were still carefully selected. For instance, our censoring range thresholds are similar to the range of the observed change (−75 to 68%). Further, whilst we don’t have a clear definition for what an increasing or decreasing population looks like (is it 1% or 10%), we can be confident that increasing and decreasing populations will fall above and below 0%, respectively. The stable category is most vulnerable to subjectivity, and so without clear definitions, we set a large range e.g. the maximum and minimum value we considered could be plausibly called stable was 5% and −5%, respectively.

Many of the qualitative and short-term (brief monitoring period) quantitative records address known data biases as they occur in less-well represented regions, species, and time-periods (Fig. 1). However, these lower quality records can be more prone to error. As a result, we developed a weighting term within the model to inflate uncertainty around trends derived over a short timeframe, with few abundance observations, and less robust methods—see Supplementary: Modelling—Weighted error.

#### Covariates

Prior to modelling, we identified missing values in some covariates (e.g. some species were missing Maximum longevity values), which can be problematic for inference if ignored^34^. We used imputation approaches^38,39^ to predict these missing values and recorded the associated imputation uncertainty alongside these predictions. Within our model, we accounted for uncertainty in the imputed estimates by treating imputed values of the covariates as distributions instead of point estimates. Specifically, for each imputed value we assigned a normal distribution defined by the mean and standard deviation of the imputed estimates. This approach allowed us to capture imputation uncertainty and improve inference robustness.

With 16 covariates and a further seven interactive effects (23 effects in total), we were conscious of overparameterizing the model. As a result, we split these parameters into three groups: (1) core parameters—which included main effects that were considered likely drivers of population change; (2) optional parameters—which included main effects we considered interesting but with little evidence to-date of any influence on trends; and (3) interaction parameters—which includes the seven proposed interaction terms. We included our core parameters (Change in human density, Primary land loss, Population area, Body mass, Change in extreme heat, Governance, and Protected area coverage) in every model, but used Kuo and Mallick variable selection^40^ to identify parameters from the optional and interaction groups that improve model fit whilst balancing the risk of overfitting.

#### Random intercepts

We used a hierarchical model structure to account for phylogenetic and spatial non-independence in the data, including species as a random intercept nested with genus, and country as a random intercept nested within sub-regions, as defined by the United Nations (https://www.un.org/about-us/member-states).

#### Model running

We ran the full model through three chains, each with 150,000 iterations. The first 50,000 iterations in each chain were discarded, and we only stored every 10th iteration along the chain (thinning factor of 10). We opted for a large chain and burn-in due to the model complexity, and to allow a broad selection of parameter combinations to be tested under variable selection. We assessed convergence of the full model on all parameters monitored in the sensitivity analysis, as well as the model intercept, and all 23 main and interactive effect slope coefficients. We checked the standard assumptions of a mixed effect linear model (normal residuals and heterogeneity of variance), and tested the residuals to ensure there was no spatial (Moran’s test) or phylogenetic (Pagel’s lambda) autocorrelation. We also conducted posterior predictive checks to ensure independently simulated values were broadly reminiscent of model predicted values.

We report the median slope coefficient and associated credible intervals for each of the main and interactive effects, and produce marginal effect plots for a selection of important parameters. These marginal effects hold all other covariates at zero (which is the equivalent of the mean, as covariates were z-transformed).

#### Limitations

Developing macro-scale models of population change is challenging as response data are biased^41^ and hard to summarise^42^, and response-covariate relationships are likely complex and numerous^2^. Within our workflow, we attempted to address these challenges, and overall, this allowed us to achieve a moderate model fit (conditional *R*^2^ ~ 0.4). We minimised biases in the trend data by integrating qualitative trends with quantitative estimates, which allowed us to increase the taxonomic and spatial scale of the work. However, biases are likely still present to some extent. For instance, whilst we have population trend data covering the full parameter space of our most influential variable (change in human development), we have more population trends in high human development countries (Supplementary Fig. 20)—given these biases, caution should be used when interpreting results. While we could not avoid some biases, we found inference was similar across different fragments of the data and model structures (Supplementary results: Sensitivity analysis). We also attempted to capture complexity by covering a more comprehensive array of covariates than many previous analyses, but we still lack data on likely important aspects that are cryptic and difficult to measure (e.g. poaching, persecution, culling, and the conservation benefits of being flagship species). Further, there are temporal lags between disturbance-events and observable changes in the population^10^ and we tested several to incorporate the lag that maximised model fit. However, it is possible that responses to different types of disturbance (e.g. habitat loss and climate change) have different lags, although this has not been quantified. Long lags (the maximum lag we explored was 10-years) may also occur and be associated with slow recoveries, but an absence of longer temporal extents in the response and covariate data largely prohibits this analysis at global scales (long temporal extent data is less available outside of the global north).

### Counterfactual scenarios

To explore how observed changes in land-use, climate and human development have influenced population trends, we developed three counterfactual scenarios, where we compared observed population change to predicted population change if habitat, climate, and human development remained static. For instance, in the climate change counterfactual scenario, we predicted each population trend using the global model (all covariate parameters) with available covariate data (e.g. land-use, governance and trait covariates), as well as taxa and location data (to provide sensitivity to the models varying random intercepts), but set the climate change covariate data to zero (in this case, change in extreme heat and change in drought). We then subtracted these counterfactual predictions from the observed trends to define ‘Difference in annual rate of change (%)’, whereby a positive value indicates carnivore populations would be in better shape (fewer declines) under the counterfactual scenario, and vice-versa. We summarise counterfactual scenarios by reporting the median Difference in annual rate of change and 95% quantiles across the observed 1123 populations.

### Socioeconomic development and non-linearity in carnivore trends

Given the large effect of human development change on carnivore population trends within our counterfactual scenarios, we further explore the potential impacts of human development change (i.e. changes in the socioeconomic standards of society) on the dynamics of potential carnivore abundance change. Specifically, we test how changing the rate of human development growth of a hypothetical low human development country could impact carnivore abundances. We test this by simulating time series of human development change between the years 1960 and 2020 along three common development pathways for low human development countries, each given: (1) a mean rate of change in human development (%) defined as Slow (1.25%), Moderate (1.5%) and Fast (1.75%); (2) a shared deceleration rate set to −0.02% per year—a key feature of the human development data is that as human development grows, its growth rate decreases; and (3) a shared initial human development value which we set as 0.2 (a hypothetical low human development country) at year 1960 (Fig. 4a). All our selected parameter values are representative of the human development data (Supplementary Fig. 2), with the Moderate pathway being largely typical for a country with an initial human development value of 0.2, while Slow and Fast represent plausible extremes.

We then used our fitted model (Fig. 2) to evaluate how the three pathways of Change in Human development would affect annual abundance of a hypothetical carnivore. This involved predicting the annual rate of change in abundance using the Change in human development pathways and the marginal effect of the Change in human development parameter from the fitted model—setting all other covariates in the model to zero, which in our z-transformed variables represents the mean. We then used the predicted annual rates of change in abundance to project carnivore abundance up to the year 2020, from an arbitrary baseline abundance of 100 in the year 1960 (Fig. 4c). These projections capture the 95% credible intervals around the human development change model coefficient, and assume constant and average values for all other effects (e.g. primary habitat loss or climate change).

### Reporting summary

Further information on research design is available in the Nature Portfolio Reporting Summary linked to this article.

## Supplementary information

Supplementary Information

Reporting Summary

## Data Availability

All data is openly accessible subject to licence conditions. Trend data was sourced from CaPTrends (https://onlinelibrary.wiley.com/doi/full/10.1111/geb.13587) and the Living Planet (https://www.livingplanetindex.org/data_portal). Covariate data: climate (https://chelsa-climate.org/), land-use (https://luh.umd.edu/), governance (https://databank.worldbank.org/source/worldwide-governance-indicators), human development (https://hdr.undp.org/data-center/human-development-index), PanTHERIA traits (https://esajournals.onlinelibrary.wiley.com/doi/10.1890/08-1494.1), AnAge traits (https://genomics.senescence.info/species/index.html), and protected areas (https://www.protectedplanet.net/en/thematic-areas/wdpa). All data is described in Supplementary Table 2, with extended descriptions of how to access and use data within the code^43^. Source data are provided with this paper.

## Source data

Source data

## Code availability

All code is available at Zenodo^43^.
