# Supplementary material for: Socioeconomic factors predict population changes of large carnivores better than climate change or habitat loss
Source: Nat Commun. 2023 Jan 24;14:74. doi: 10.1038/s41467-022-35665-9 (PMC9873912; doi:10.1038/s41467-022-35665-9)
Supplement: Supplementary file 1 — Supplementary Information [file 41467_2022_35665_MOESM1_ESM.pdf]

Socioeconomic factors predict population changes of large carnivores better than climate change or habitat loss – Supplementary Information

Supplementary Methods

Population trends

We sourced population trend information for all species in the families Canidae, Felidae, Hyaenidae, and Ursidae of the order Carnivora from two large trend datasets: CaPTrends [1] and the Living Planet Database [2]. CaPTrends contributed 1,220 trends, and the Living Planet Database contributed 350, combining to produce a cumulative 1,474 unique (non-duplicated) trends. In the Living Planet Database, and for most records in CaPTrends, trends are reported as a time-series of abundance (or density) estimates. We modelled these time-series with log-linear regressions, where abundance (the response) was log_e transformed, and year of abundance estimates was selected as the predictor. We added 1% of the maximum abundance value to all abundances in each time-series to handle cases where abundance equaled zero and could not be log transformed. We included a continuous Ornstein-Uhlenbeck autoregressive process to control for temporal autocorrelation in these models, where abundances are more similar when near in time. We extracted the slope coefficient which represents the annual instantaneous rate of change, sometimes called the population growth rate (r_t). There are also other formats of quantitative trends in CaPTrends which fall into three broad data types, all of which we converted into an annual instantaneous rate of change (r_t):

1) Finite rate of change

$$r_t = log_e(\lambda)$$

Where λ represents the mean annual finite rate of change.

2) Estimates of relative abundance change between two points in time (e.g. percentage or fold change in the past 10 years)

$$r_t = \frac{log_e(1 + (P/100))}{N}$$

Where P represents the additive percentage change (e.g. a population doubling in size = 100%), and N is the difference in time (in years) between the two estimates of abundance. For fold changes, we first converted the fold change into an additive percentage change.

3) time-series of population change estimates, reported as either population lambdas or percentage changes e.g. in year 1 the population doubled ($\lambda = 2$) and in year 2 it halved ($\lambda = 0.5$). We back-converted the change estimates into abundance estimates against a constant value of 100. We then fitted log-linear regressions with abundance and year, as in the abundance time-series.

We converted all annual instantaneous rates of change into an annual rate of change percentage to improve interpretability. These rates of change ranged from -75% to 68%, but the majority of values fell within -10% to 10% (Figure S1a). Alongside the quantitative records, 138 populations in the CaPTrends dataset were only described qualitatively with categories: Increase, Stable, and Decrease. Population trends were assigned into these categories based on their description in the primary literature i.e. if a population was solely described as stable by the authors in the primary literature, without any form of quantification, we recorded the population as Stable. As such, we don't know what levels of abundance change would be needed to meet each category as each primary source had their own subjective definition which was not made explicit in writing.These records were more common for populations located in traditionally poorer-sampled countries (e.g. with lower human development), so whilst they are less informative (only describing the direction and not the magnitude), we deem them important to reduce known biases [1]. As a result, we used a combination of annual rate of change (%) and qualitative categories as our response in our inference model – see below. Despite including both types of trend, our data still contained many of the same spatial and temporal biases that plague macro scale biodiversity change studies (Figure 1).

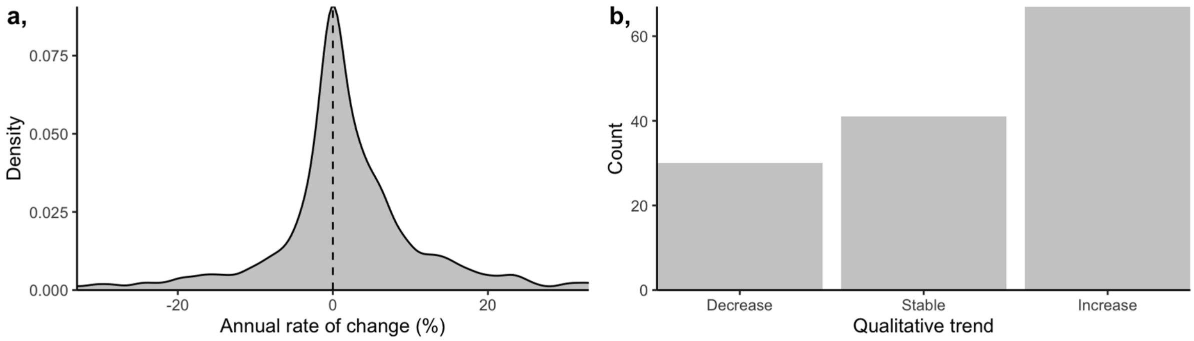

Figure S1. Distribution of quantitative (a; N = 985) and qualitative (b; N = 138) trends for the 50 large carnivore species with available data.

Covariates

We extracted a variety of covariates that we considered potential drivers of population change in large carnivores (Table S1). The data used to derive these covariates is described in Table S2. Our covariates fall into four categories: land-use, climate, governance, and traits (Figure 1). One of the challenges in identifying how covariates impact population trends is matching the spatial scale of the covariate with the population i.e. how much of the population is affected by the covariate. To tackle this problem, we used data on the area of extent of each population to generate a circular distribution zone around the population's coordinate centroid. We refer to this as the 'population area' hereafter. In populations without a reported extent (N = 347), we searched the locality and location description online to identify the approximate size of the population area. For example, the top result in a Google search for 'Serengeti area' described the location as 30,000km^2. In some cases, we were able to find a location on a map, but its area was not described in any of the Google search results. To handle cases like this, we assigned the population to one of the following categories: small locations (e.g. towns and counties): 1,000km^2 [N = 123], medium locations (e.g. regions and states): 10,000km^2 [N = 151], and large locations (e.g. countries): 100,000km^2 [N = 73]. For example, one trend was located in an area called 'Thekwane, Kruger National Park, South Africa', which from a Google search appeared to be a small section of Kruger National Park. As the area of Kruger is estimated at 20,000km^2, we opted

to label Thekwane as a small location (1000km^2). These categories were arbitrarily chosen with the purpose of trying to compile covariate data at an appropriate spatial scale.

Table S1. Proposed direction of effect and rationale for inclusion for each of our 16 main effects and 7 interactive effects. For 'Effect', plus and minus signs indicate that we expect populations to increase or decrease, respectively, as the parameter value increases. An 'Effect' value of zero would indicate that we don't expect a simple main effect, and the variable is only included to allow testing of interactive effects, or to control for features in the data (e.g. the spatial size of population). The rationale is a brief description of why we expect a given effect.

Parameter	Effect	Rationale
Land-use		
Primary habitat loss	-	Habitat loss could cause declines in food availability and denning habitat, leading to population declines. Habitat loss could also indirectly cause more human interaction which may lead to population declines e.g. persecution, retaliatory killings etc.
Change in human density	-	High human density is likely associated with more human-carnivore interactions, which could increase persecution and retaliatory killings.
Change in natural land	+	Increased natural land could improve food availability and denning habitat.
Primary habitat loss * Ecological niche breadth	+	Species with a higher ecological niche breadth could be more resilient to habitat loss
Primary habitat loss * Change in human density	-	When primary habitat is lost, we would expect the impacts on carnivores to be most extreme when replaced by human dominated landscapes
Primary habitat loss * Change in natural land	+	When primary habitat is lost, we would expect the impacts on carnivores to be least extreme when replaced by natural (secondary habitat) landscapes
Traits		
Population area	0	We don't expect any directional main effect with population area. Variable only included to control for the unknown effects of population area.
Body mass	0	We don't expect any directional main effect with body mass. Variable only included to test interactive effects.
Reproductive output	+	Faster breeding species have a greater capacity for recovery
Maximum longevity	+	Longer-lived species are less likely to present with declines as they can persist for longer e.g. extinction lag
Climatic niche breadth	0	We don't expect any directional main effect with climatic niche breadth. Variable only included to test interactive effects. A positive effect here could be evidence of high climatic niche breadth species persisting under climatic threats that we have not captured.
Ecological niche breadth	0	We don't expect any directional main effect with ecological niche breadth. Variable only included to test interactive effects. A positive effect here could be evidence of high ecological niche breadth species persisting under environmental threats that we have not captured.
Climate		
Change in extreme heat	-	We expect population trends to decline under extreme heat, as species, communities and habitats become stressed and decline - community turnover and transition
Change in drought	-	We expect population trends to decline under drought, as species,

		communities and habitats become stressed and decline -community turnover and transition
Change in extreme heat * Body mass	-	We expect larger bodied species to decline at a faster rate, as larger bodied species could experience greater thermal stress
Change in extreme heat * Protected area coverage	+	We expect populations inside protected areas to be more resilient to rising temperatures
Change in extreme heat * Change in drought	-	We expect a synergistic effect between heat and drought, where populations in the hottest and driest conditions will decline faster.
Change in extreme heat * Climatic niche breadth	+	We expect species with a greater climatic niche breadth to be more resistant to the climate induced population declines.
Governance		
Protected area coverage	0	Populations in protected areas are expected to be stable. A positive effect here could mean declines outside protected areas exceed those within. A negative effect could indicate that population growth is greater outside protected areas.
Governance	+	High governance is associated with carnivore population growth, as high governance countries have: 1) lower population baselines in the available data; and 2) effective rule of law, management of corruption, and species protection will promote population growth.
Change in human development	-	Rapid growth in development will lead to population declines as the growth results in societal changes e.g. shift to industrialisation, urbanization, reduced tolerance etc.<br><br>Note: We only study the change in human development and not governance, as governance is relatively static over the studied timeframes.
Human development	+	High development is associated with carnivore population growth, as high development countries have: 1) lower population baselines in the available data; and 2) improved quality of life promotes tolerance with carnivores.
War present	-	War results in poaching and degradation of habitat leading to population declines

Table S2. Description and citation of data sources used to derive covariates.

Category	Data source	Description
Land-use	Land-use harmonization 2 (LUH2)[3]	Model based estimates of 11 land-use categories, ranging from primary vegetation to urban land-uses. Each pixel (resolution: 0.25 degrees) within the global extent describes the proportional representation of each of the 11 land-use types at a given point in time. This dataset is available for all years from 1850 - 2100 with annual time steps.<br><br>This dataset is used in the creation of the 'Primary land loss' and 'Change in natural land' covariates.
Land-use	Global human settlement - population (GHS - POP) [4]	Model based estimates of human population counts across a global extent, with a pixel resolution of 0.1 degrees. This dataset is available from 1975 - 2030 in five year time steps.<br><br>This dataset is used in the creation of the 'Change in human density' covariate.
Climate	CHELSAcruts[5]	Earth system model based estimates of monthly maximum temperature, minimum temperature, and precipitation, reported across a global extent at 0.008 degree resolution. This dataset covers the time frame 1901 - 2016<br><br>This dataset is used in the creation of 'Change in extreme heat' and 'Change in drought'
Climate	CHELSA V1.2 [6]	As above, but also reports monthly mean temperature and only covers 1979 - 2013. This dataset is solely used to correct mid-point temperatures derived in the above dataset (see Covariates: Climate).<br><br>Used in the 'Change in drought' covariate
Governance	UCDP-PRIO [7]	The UCDP/PRIO Armed conflict dataset lists conflicts (between 1946-2019) where fatalities per year exceeded 25 and at least one of the parties was governmental. This dataset is global in extent<br><br>Used in the creation of 'War present' covariate
Governance	World governance indicator [8]	This dataset contains six governance metrics that combined create a governance score. This score is available for the majority of countries and is measured annually, ranging from 1996 - 2020.<br><br>Used in the creation of the 'Governance' covariate
Governance	UN human development index [9]	This dataset describes the human development index scores for the majority of countries, is measured annually, and ranges from 1990 to 2020.<br><br>Used in the creation of the 'Human development' and 'Change in human development' covariates
Governance	World database of protected areas [10]	This dataset describes the spatial distribution of protected areas from 1960-2020, with protected areas represented by a series of polygons, with annual temporal resolution.<br><br>Used in the creation of the 'Protected area coverage' covariate.
Traits	IUCN range maps [11]	This dataset describes best estimates of species current (year varies depending on the date a species is assessed; varies from 2015 - 2021) and historical range/distribution.<br><br>Used in the production of the 'Climatic niche breadth' covariate
Traits	IUCN habitat classifications 3.1 [12]	This describes best estimates of habitat types each species occurs in, or has occurred in. These current IUCN habitat scheme has 18 categories, each with multiple sub-categories.<br><br>Used in the production of the 'Ecological niche breadth' covariate
Traits	WorldClim 2.1: BioClim [13]	This dataset describes 19 bioclimatic variables (e.g. mean temperature) across a global extent (Resolution: 0.008 degrees), each averaged over a thirty year period (1970 - 2000).<br><br>Used in the production of 'Climatic niche breadth'
Traits	PanTHERIA [14]	This dataset describes a large array of species-level trait values (e.g. body mass)<br><br>Used in the production of a variety of trait covariates

Traits	AnAge [15]	This dataset describes a large array of species-level trait values (e.g. body mass)<br>Used in the production of a variety of trait covariates

Given population areas regularly exceeded 10,000km^2 (Figure S2a), it was not computationally feasible to extract covariates over the entire area; thus, we sampled from a random selection of points within each population area, sampling more frequently in larger areas (range: 13 – 295 sampling points, Figure S2b). Random sampling was only used for land-use and climate covariates, as governance covariates are measured at the national level, and all traits (except for population area itself) represent species level averages. The population areas and corresponding sampling points were defined with a Mollweide equal-area projection, but we transformed areas and points back into a WGS84 projection to match all covariate rasters (see below). In all covariates, 'population monitoring period' refers to the period (start and end year) the population was monitored for.

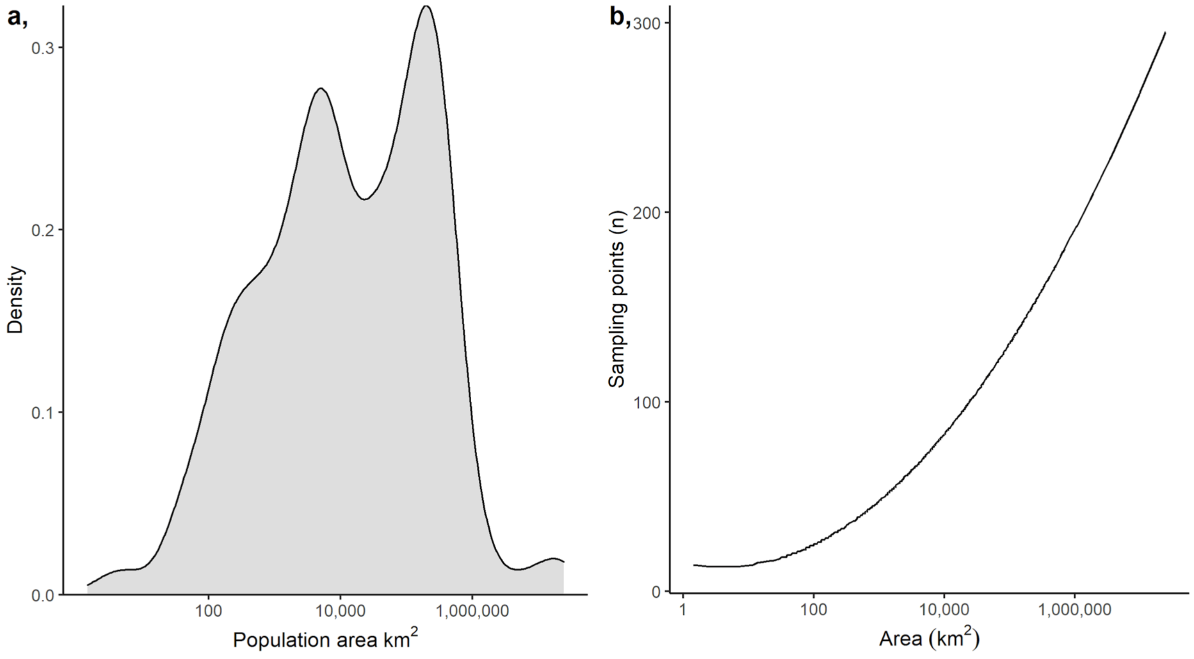

Figure S2. a) Distribution of population areas, the area of extent of population monitoring for 1,474 population trends extracted from CaPTrends [1] and the Living Planet Database [2]. b) Frequency of covariate sampling points relative to population area size, where populations occurring over larger areas receive more covariate sampling. Both x-axes are on a $\log_{10}$ scale.

Land-use

We extracted three land-use covariates: Primary habitat loss, Change in natural land, and Change in human density. Primary habitat loss and Change in natural land were derived from the land-use harmonization dataset (Table S2), which reports the annual proportional coverage of 11 land-use types between 1850 and 2015AD, at a 0.25° spatial resolution. To make the land-use types more biologically relevant to predators, we amalgamated a selection of the 11 types into two summary-types: primary habitat – the sum of 'forested primary' and 'non-forested primary'; and natural land – the sum of 'potentially forested secondary', 'potentially non-forested secondary', 'managed pasture' and 'rangeland'. To estimate Primary habitat loss we found the mean primary habitat across sampling points in each population area for each year in the population monitoring period. We then estimated the rate of loss in primary habitat over time by dividing the rate of loss in each year by the previous year, and then converted this to a percentage loss. We defined the mean Primary habitat loss (%) for each population area as the average across this time-series of loss rates. We followed an identical procedure for Change in natural land. Importantly, our estimate of Change in primary habitat could only decrease or remain stable, whilst natural habitat can fluctuate up and down.

We estimated the Change in human density using the Global human settlement human population raster (Table S2), which describes the human density per km^2 for four years: 1975, 1990, 2000, 2015. For each year, we reduced the spatial resolution to 0.1° by averaging (mean) over finer-resolution pixels. In order to estimate the Change in human density for each population area's monitoring period, we had to estimate missing human density values (years) in each population area. To do this, we first extracted the mean human density across each population area, in all four of the available years. In each population area, we then used a log-linear regression to model human density (base log transformed) against year, and then predicted human density between 1960 and 2015. We modelled year (predictor) with a cubic fit because human density was non-linear. We then extracted the back-transformed predicted values of human density for all years in each population area. As we were only working with four data points, model predictions were highly uncertain. This uncertainty was included by resampling our model with 100 bootstrap iterations. For each population monitoring

period and iteration, we extracted the predicted human densities and estimated the rate of change (%) as calculated for the other land-use covariates. Finally, we calculated the mean human density rate of change (%) across all iterations, as well as the standard deviation, which was used to represent uncertainty in the values within the inference model (see below).

Climate

Our two climatic covariates, Change in extreme heat and Change in drought, describe how the number of months exceeding an extreme heat or drought threshold (respectively) changed between a pre-industrial period (1901 – 1920) and the population monitoring period (variable time periods between 1960 and 2015). In previous work looking at the link between climate change and population abundance change, it has been common to study the change in the average temperature or precipitation over time like in [16], instead of extreme events. However, we opted to instead focus on extreme events as we suspected they were more likely to represent direct mortality risk (e.g. exceeding thermal maxima) for species than changes in average temperatures or precipitation.

To derive the Change in extreme heat covariate, we compiled a raster time-series of monthly maximum temperature from 1901 to 2015, at a 0.008° resolution (Table S2). From this, we calculated the mean and standard deviation of the monthly maximum temperature in the pre-industrial period (1901 - 1920), and defined the extreme heat threshold as the mean plus 2 standard deviations of the mean in each pixel. With this approach, approximately 2.5% of the monthly values in the pre-industrial period would exceed this threshold, thus, representing rare extreme heat events. Next, we quantified the actual mean number of months per year in the pre-industrial period that exceeded this threshold, as well as the number of months to exceed this threshold in all years between 1960 and 2015 for every pixel. We then subtracted the number of threshold-exceeding months in each year (1960-2015) from the mean threshold-exceeding months in the pre-industrial period, creating a raster time-series describing the difference in threshold-exceeding months. This value could inform us about how the number of months exceeding the threshold in a given year in which a carnivore population was studied differ to the average across the pre-industrial period. Finally, for each sampling point in

each population area, we found the mean difference across the population monitoring period, and then averaged this difference across all sampling points to produce a population area estimate of the Change in extreme heat.

To derive our Change in drought covariate, we required two raster time-series describing the mean monthly temperature and precipitation, both from 1901 to 2015. Whilst the monthly precipitation data (total rainfall in mm) was readily available (Table S2), we had to derive a proxy for the mean temperature data which required two steps. First, we used the monthly maximum and minimum temperatures from CHELSAcruts [5] to derive a raster time-series of midpoint temperature values from 1901 to 2015. Then, we used a linear regression of the mean monthly temperatures, which were available from CHELSA V1.2 [6] a subset of years (1979-2013), against the midpoint monthly temperatures to determine how midpoint values could be corrected to represent a proxy of mean values. This regression was nearly a 1:1 relationship with near perfect fit (R-squared = 0.99). Using our proxy mean temperature, we calculated Thornthwaite's evapotranspiration across the raster time-series, which uses the mean temperature, latitudinal position and number of daylight hours to estimate the evapotranspiration rate [17]. Next, we subtracted this monthly evapotranspiration estimate from the monthly precipitation estimate to produce Thornthwaite's standardised precipitation-evapotranspiration index (spei), a standard metric used to describe water availability [18]. We then proceeded to estimate a spei threshold and the mean difference in months overlapping the threshold (pre-industrial vs. population monitoring period) in an identical way to how monthly maximum temperature was used to estimate the Change in extreme heat covariate.

Governance

From the literature, we identified five governance covariates that we considered important to large predator population trends, four of which were measured at the country-level: War present [19], National governance [20], Human development [21], and Change in human development. We used three datasets to populate these covariates: 1) For War-present, we used the UCDP/PRIO Armed conflict dataset (Table S2), which lists conflicts (between 1946-2019) where fatalities per year exceeded 25 and at

least one of the parties was governmental. We summarised this dataset into a time-series that describes whether a war was taking place in each country's territory in each year between 1960 and 2016. 2) For Governance, we extracted the world governance indicator metrics (Table S2), which presents six annual governance time-series for each country between 1996 and 2016. 3) Finally, for Human development we sourced the UN human development index (Table S2), which provides an annual time-series describing life expectancy, education level, and income per capita between 1990 and 2016 for 189 countries.

As the governance and human development indicator data only stretch back until 1996 and 1990, respectively, some of the trend data (22% and 7%, respectively) preceded the indicator values. We imputed missing values through a multiple imputation chained equations (MICE) framework [22]. We used a hierarchical normal (2l.pan function) imputation model, which is comparable to a linear mixed model regression, where observations are nested into the different countries, with the following fixed effects: the year of the observation, the six governance indicator metrics, the human development index, whether war was present in that year (yes or no), as well as the country's gross domestic product (log 10 transformed). MICE imputations are stochastic and repeated numerous times, creating an approximate distribution for each missing value. We imputed missing values for each variable between 1960 and 2016 in each country, and repeated the imputation 100 times with a 50 iteration burn in – all variables showed convergence (Figure S3).

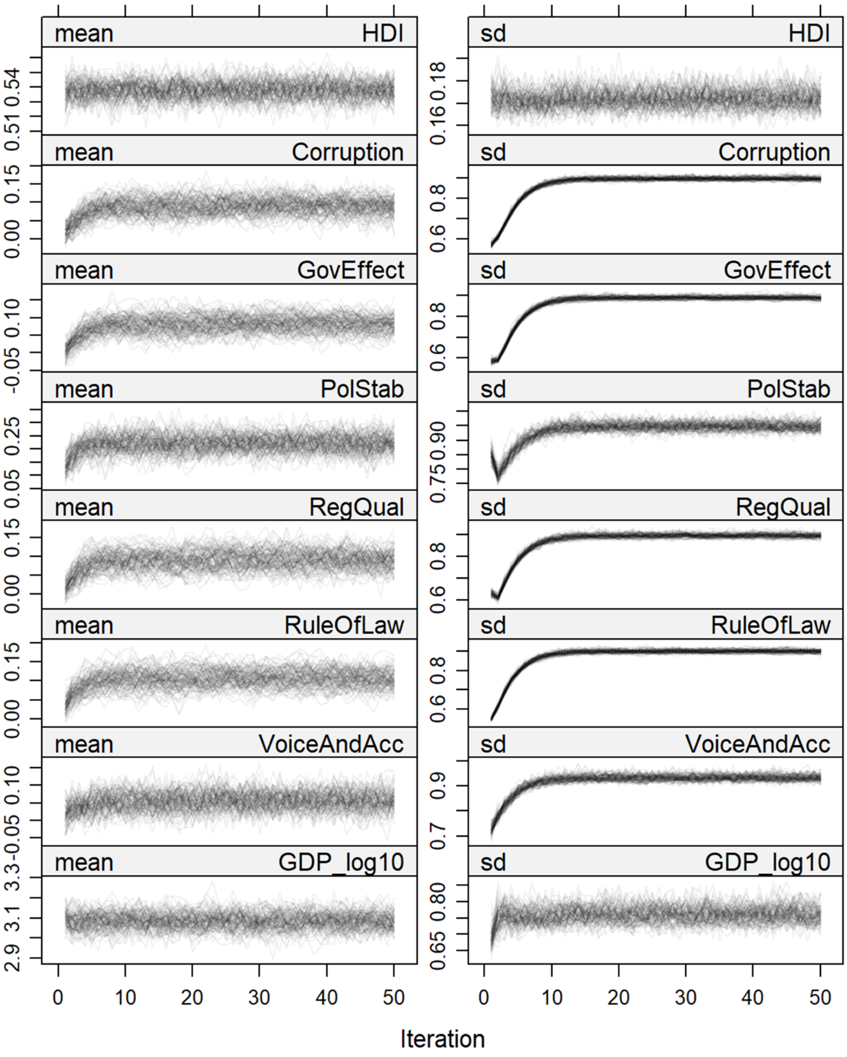

Figure S3. Convergence of mean (left) and standard deviation (right) of variables with missing values in the imputation model: HDI – human development index, Corruption – control of corruption, GovEffect – government effectiveness, PolStab – political stability and absence of violence, RegQual – regulatory quality, RuleOfLaw – rule of law, VoiceAndAcc – voice and accountability, and GDP_log10 – gross domestic product (log 10 transformed). Convergence ran with 50 iterations and 100 chains.

Using the imputed datasets, we extracted the mean value across the six governance indicators in each country, year, and imputation chain. We then calculated the mean and standard deviation of this combined governance across the imputation chains to produce an annual governance time-series (and associated error) for each country. For Human development, we averaged over the 50 stored imputation chains to calculate the mean and associated standard deviation for each country and year. We ensured imputed values fell within the natural constraints of the data (e.g. within the range of 0-1 for human development) and connected seamlessly with the observed human development and governance values (Figure S4). For governance, there is no way of knowing if imputed values are

accurate, but for human development, certain countries have estimates of 'historical index of human development (HIHD)' [23], which whilst calculated slightly differently, still represent a reasonable proxy of human development change pre-1990. We compare our imputed estimates of human development to the HIHD estimates in Figure S5, where in the majority of cases, HIHD estimates fall within the confidence intervals of our imputed estimates, even capturing complex non-linearity like that observed in Botswana (BWA), and to some extent Rwanda (RWA). Across all countries (including the nine displayed in Figure S5), our imputed values (human development values pre-1990) are highly correlated (Pearson correlation = 0.925 across all countries) with the HIHD estimates, indicating that generally our imputed estimates are highly synchronous and likely capturing any non-linearity in the HIHD estimates. We explicitly capture our imputation uncertainty to deal with error introduced by imputation (see *Modelling - Inference model* below).

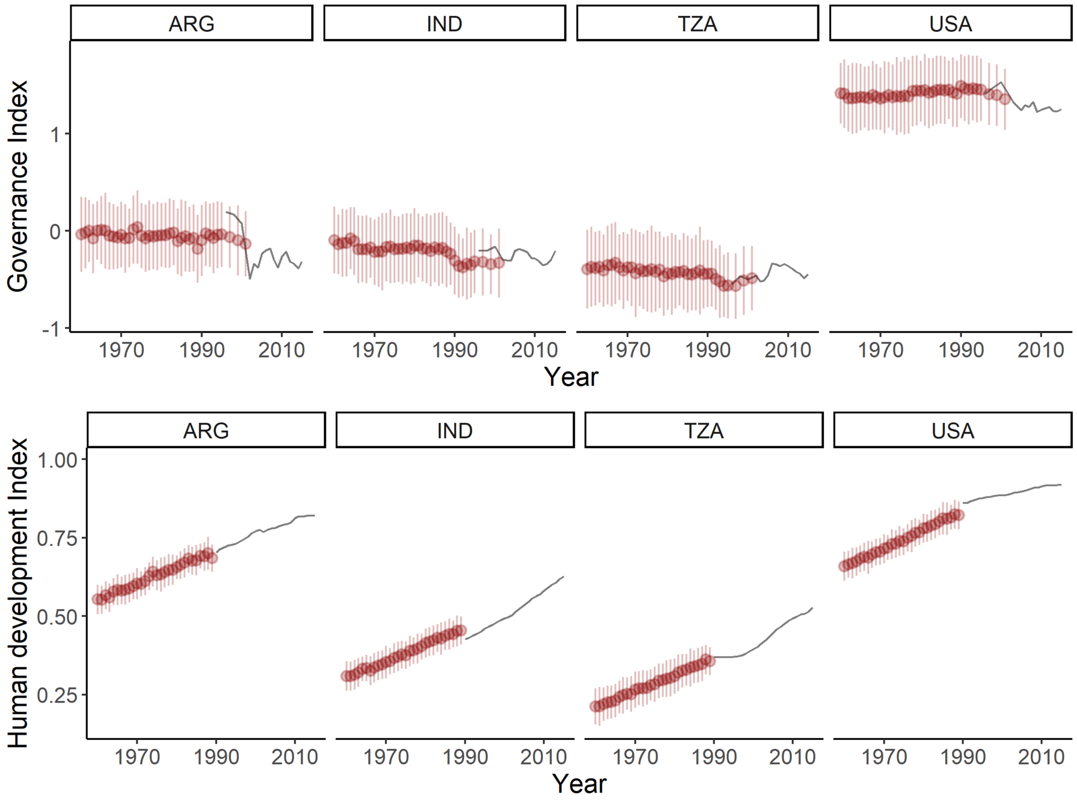

Figure S4. Governance (top) and human development (bottom) index scores for Argentina (ARG), India (IND), Tanzania (TZA), and the United States of America (USA). True values are depicted with the black line, whilst the mean imputed values (point) and associated 95% confidence intervals (bars) are depicted in red; derived from the 50 imputation chains.

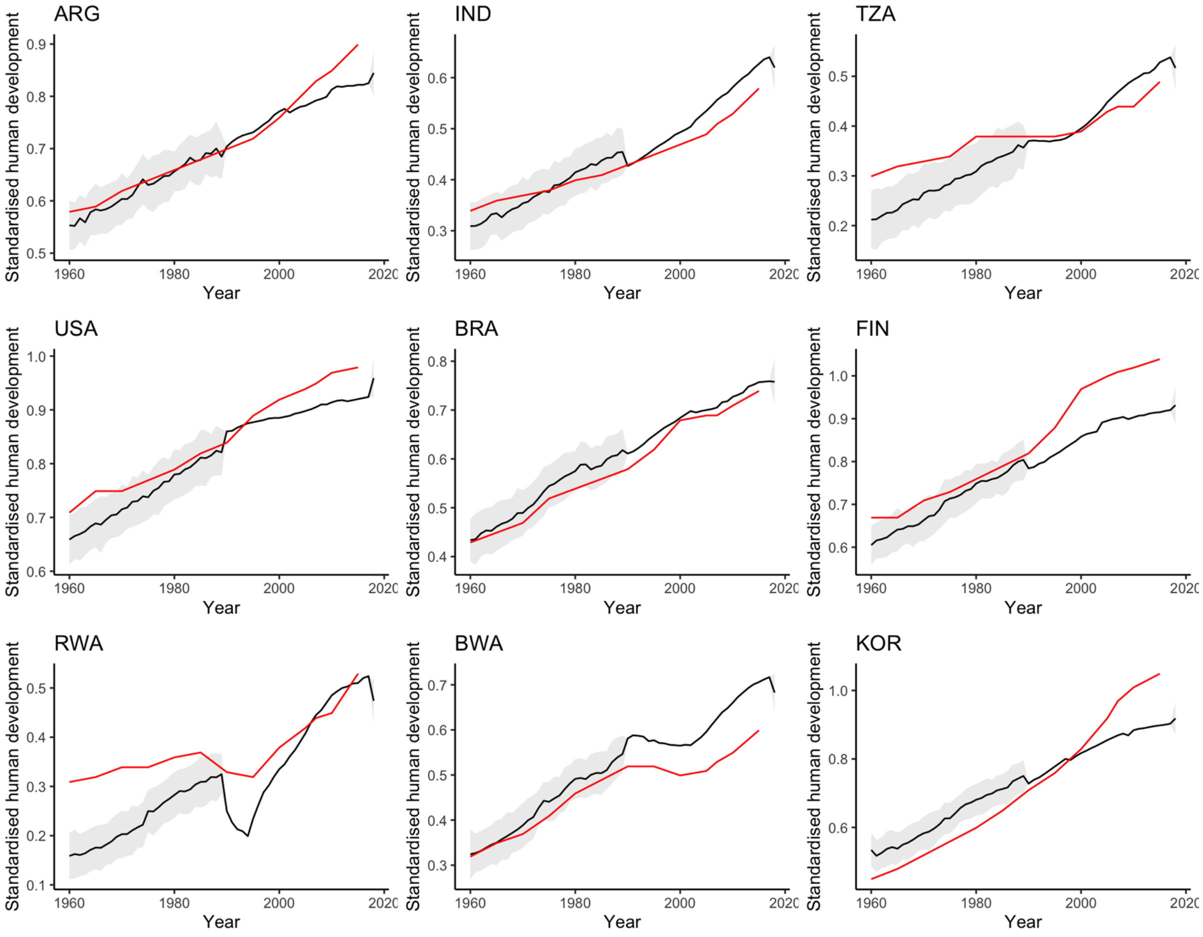

Figure S5. Human development (black) and historical index of human development (HIHD; red) time-series for a selection of 9 countries covering a broad economic and spatial extent. Grey shading represents the estimates of uncertainty around imputed human development values, and the solid line within this shading represents the median imputed estimate. Despite human development and HIHD both ranging between 0 and 1, they can not be compared directly. For example, whilst the USA has similar trend in both datasets, the raw values differ (human development is 0.9 whilst HIHD is 0.78), with the mean in the USA fragment of the human development dataset 0.11 greater. To address these different means in each country and aid direct comparison between the datasets, we adjusted the mean of HIHD dataset, by adding the difference between the two datasets to the HIHD dataset, per country.

After we derived the governance and human development time-series, we began extracting the covariates. For War present, we created a binary variable that described whether war(s) had occurred in the country where the population is located, at any point during the population monitoring period. For Governance and Human development, we extracted the mean scores per country, and associated standard deviations from the first year of the population monitoring period. For Change in human development, we extracted all human development values across the population monitoring period, and divided each value by the value in the previous year to produce a time-series describing the

annual changes in human development. We then averaged these values and converted the average into a percentage which describes the annual rate of change (%) in human development.

Our only governance covariate not measured at the country-scale is Protected area coverage. For this variable, we compiled the annual time-series of all terrestrial protected areas polygons covering the period 1960 to 2020 from the World Database of Protected Areas [14]. In each year, we converted the polygons into a 0.1° resolution raster describing the proportional cover of protected areas in each pixel. In the final year of the population monitoring period, we calculated the mean coverage of protected areas across pixels within the population area.

Traits

We identified five species traits which could influence population trends in large predators: Body mass, Maximum longevity, Climatic niche breadth, Ecological niche breadth, and Reproductive output. Body mass describes the mean body weight of an adult of the species in grams ($\log_{10}$ transformed), Maximum longevity describes the maximum lifespan of an individual of the species in years (log 10 transformed), and Climatic niche breadth describes the standard deviation of the mean monthly temperatures (mean averaged over all years between 1970 and 2000) across the species current IUCN range, calculated using WorldClim 2.1 (Table S2). The other two trait-covariates, Ecological niche breadth and Reproductive output, are principal components of a larger array of traits. Specifically, Ecological niche breadth captures habitat and diet breadth. Habitat breadth is defined as the frequency of different IUCN habitat classifications the species occurs in (e.g. a species would be given a score of 2 if it only occurs in 'Forest: boreal' and 'Forest: sub-arctic'). Diet breadth is defined as the number of different food-types the species has been recorded consuming (or with evidence of consuming through faecal or stomach content analysis), from the following 12 options: mammals, birds, reptiles and amphibians, fish, invertebrates, fruit, pollen and nectar, leaves and branches, seeds, grass, roost and tubers, and carrion – sourced from an unpublished trait dataset [24]. Our Reproductive output trait is a principal component of the following traits (all log 10 transformed): interbirth

interval, gestation length, litter size, minimum breeding age, neonatal body mass, and weaning age. As a result, our five traits of interest were reliant on collecting values for 12 common traits.

We sourced values for our traits from three different trait datasets: PanTHERIA, AnAge, and an unpublished large predator trait dataset (Table S2). We used multiple trait datasets to populate missing values at the species level. However, the values sometimes differed between the trait datasets, and in these cases, we created multiple records for the species to capture this uncertainty in the trait value. As a result, many species had more than one value for a given trait. However, despite using multiple trait datasets, values were still missing for some species in some traits (Table S3), and so we imputed missing trait values with Rphylopars [25]. Rphylopars outperforms MICE imputation (used in the governance covariates above) when imputing species traits as it uses both the trait values and species' phylogeny to estimate missing values – Rphylopars is considered one of the best imputation methods [26]. In our Rphylopars model, we trialled three [27] Carnivora phylogenies to ensure the imputations did not drastically change depending on the phylogeny (Figure S6). Once we confirmed the choice of phylogeny had little impact, we proceeded with the rest of the analysis only using the phylogeny considered 'best' by [27]. We also included all 12 traits mentioned above in the imputation model, and six other traits to attempt to account for biases in the imputation model, specifically: species area of occurrence, minimum absolute latitude species occurs at, maximum absolute latitude species occurs at, difference in maximum and minimum latitude, maximum mean monthly temperature species occurs at, and minimum mean monthly temperature species occurs at. As the phylogenies we used were not perfectly matched to the CaPTrends and Living Planet Index taxonomies, we corrected synonymous species names in the phylogeny, and where species included in the taxonomy were absent from the phylogeny, we appended the species to a close relative node (inferred from taxonomy).

Table S3. Percentage of values missing in each trait. The six additional traits added simply to minimise bias had no missing values and so are excluded from this Table.

Trait	Missing trait values (%)
Body mass	8.0
Maximum longevity	12.6
Climatic niche breadth	0.0
Habitat breadth (part of ecological niche breadth)	0.0
Diet breadth (part of ecological niche breadth)	23.0
Age of sexual maturity (part of reproductive output)	24.1
Litter size (part of reproductive output)	10.3
Gestation length (part of reproductive output)	9.2
Weaning age (part of reproductive output)	27.6
Interbirth interval (part of reproductive output)	12.6
Neonatal body mass (part of reproductive output)	28.7

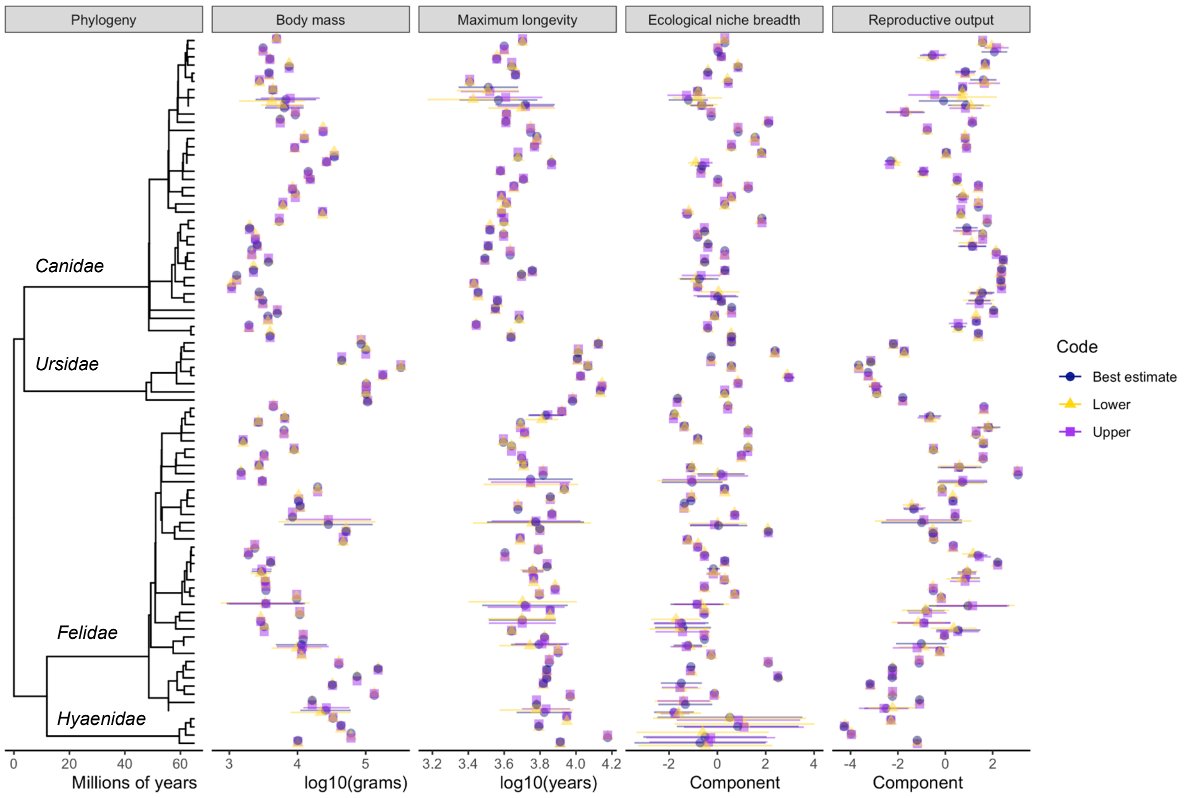

Figure S6. Trait values represented on the phylogeny; climatic niche breadth is excluded as it had no missing values. Error bars represent the 95% confidence around the mean imputed values. If observations were complete (i.e. not missing values) the standard deviation around the observation was zero and so there are no confidence intervals. We include the three phylogeny types in [27].

An advantage to using Rphylopars is that it provides an estimate of the standard deviation around the missing trait values, which is something we wanted to capture in our modelling (see Inference model below). Three of our traits were used as covariates directly within the modelling (Body mass, Maximum longevity, and Climatic niche breadth), so required no further manipulation as their

associated standard deviations were available from the imputation. However, Ecological niche breadth and Reproductive output required dimension reduction through principal component analysis (PCA), with the number of variables shifting from 2 to 1, and 6 to 1, respectively. Performing PCA on the mean values would fail to capture trait uncertainty, and so instead we developed normal distributions for each species' trait value using their mean, and an error of one standard deviation. We then sampled from each distribution 100 times, and each time conducted a PCA on the trait to develop an eigenvector. We saved the eigenvector values on each of the 100 repeats, and once the repeats were complete, we calculated the mean and standard deviation for each species across the eigenvector values. This PCA sampling procedure was performed separately on the Ecological niche breadth and Reproductive output components. We examined the trait values to ensure they were plausible (i.e. fell within the known range of trait values and trait values were similar to closely related species; Figure S5), and also checked between-trait correlations were acceptable i.e. sufficient variance in the correlation (Figure S7).

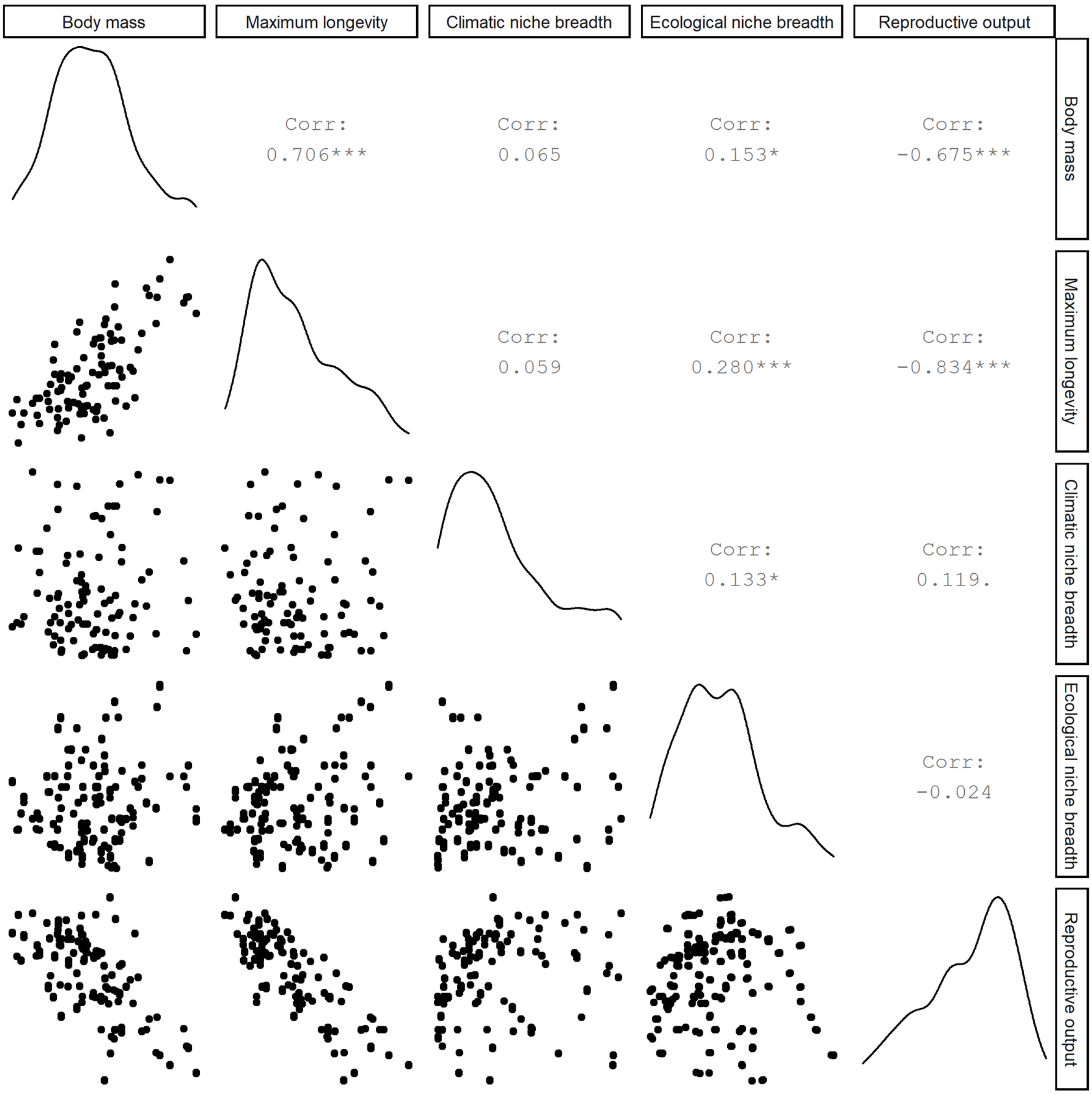

Figure S7. Distributions of traits are represented on the diagonal. Two-sided Pearson correlations between traits are represented above the diagonal with varying levels of statistical significance (p-value): '***' when p<0.001, '**' when p<0.01, '*' when p<0.05, and '.' when p<0.10. The scatter of observations (one point per species) below the diagonal graphically represents these relations.

Temporal lag

A challenge in identifying how covariates impact population trends, is matching the temporal scale of the covariate with the population i.e. How long is the lag between the covariate impact and a change in the population?. This lag period is likely variable across covariates (i.e. it could be different with land-use and climate features) and species traits. For example, recent work has suggested population change in large mammals peaks at approximately 8 years after forest loss [28]. As a result, we conduct sensitivity analysis (see Sensitivity analysis below) to determine how model fit was influenced by lag

selection, considering three options: 1) No lag, so covariate changes are measured between the start and end year of each population monitoring period. 2) Five-year lag, where covariate changes are measured between the five-years prior to the start of each population monitoring period, and run to the end of each period. 3) Ten-year lag, where covariate changes are measured between the ten-years prior to the start of each population monitoring period, and run to the end of each period.

Cleaning data

We opted to remove a selection of the population trend and covariate data as the values were deemed unreliable or unsuitable. Specifically, we removed any population trend records beginning before 1970 or after 2016 (N = 11), where governance data was largely incomplete. We also removed records overlapping multiple countries (N = 10), and any population trends with an excessively large population buffer-area (N = 40) – we set the threshold at 2 million km^2 which could accommodate state and small-country level estimates, but would exclude large countries. For example, the largest population area in the dataset covered all of Russia (~21 million km^2). Any population trends discussing non-native species were removed (N = 6), as well as records not overlapping any land (N = 4) e.g. *Ursus maritimus* populations occurring exclusively on sea-ice. We also removed any population trends where the population had either recolonised an area or become locally extinct (N = 80), which represent an extreme form of population change that could skew our inference. After excluding records, we were left with 985 estimates of annual rate of change, and 138 qualitative descriptions of change.

Modelling - Inference model

We fitted a hierarchical linear model (Figure S8) to determine the effect of a combined 23 covariates and interactive effects on the rate of change in large predator populations. Our model development falls into seven compartments: response, random intercepts, coefficients and covariates, imputation uncertainty, weighted error, confirming parameters, and model running. The model was written in BUGS language and implemented in JAGS 4.3.0 [29] via R 4.0.3 [30].

Response

The core of our model is a linear regression with fixed and random effects attempting to predict some latent state (unknown annual rates of change; Equation 1). Our observed annual rates of change are realisations of this latent state and are linked to linear model predictions through a state-space structure (Equation 2).

$$\textbf{\textit{Eq. 1}}\ \boldsymbol{r_p} = \alpha intercept + \alpha random\_intercepts + \beta 1\text{-}23$$

$$\textbf{\textit{Eq. 2}}\ \boldsymbol{r_o} \sim \boldsymbol{r_p} + \boldsymbol{e}$$

Where $\boldsymbol{r_p}$ represents the deterministically predicted latent annual rates of change from a linear model (simplified here) containing an intercept and random intercepts (α), as well as fixed effect beta (β) coefficients. $\boldsymbol{r_o}$ represents the observed (or realised) annual rates of change which are stochastically linked to a normal distribution of $\boldsymbol{r_p}$. The uncertainty in the normal distribution is determined by error e. When building the model, we identified that the model residuals exhibited a heavy tailed t-distribution and so we transformed our responses into a gaussian distribution with an inverse-hyperbolic sine transformation [31].

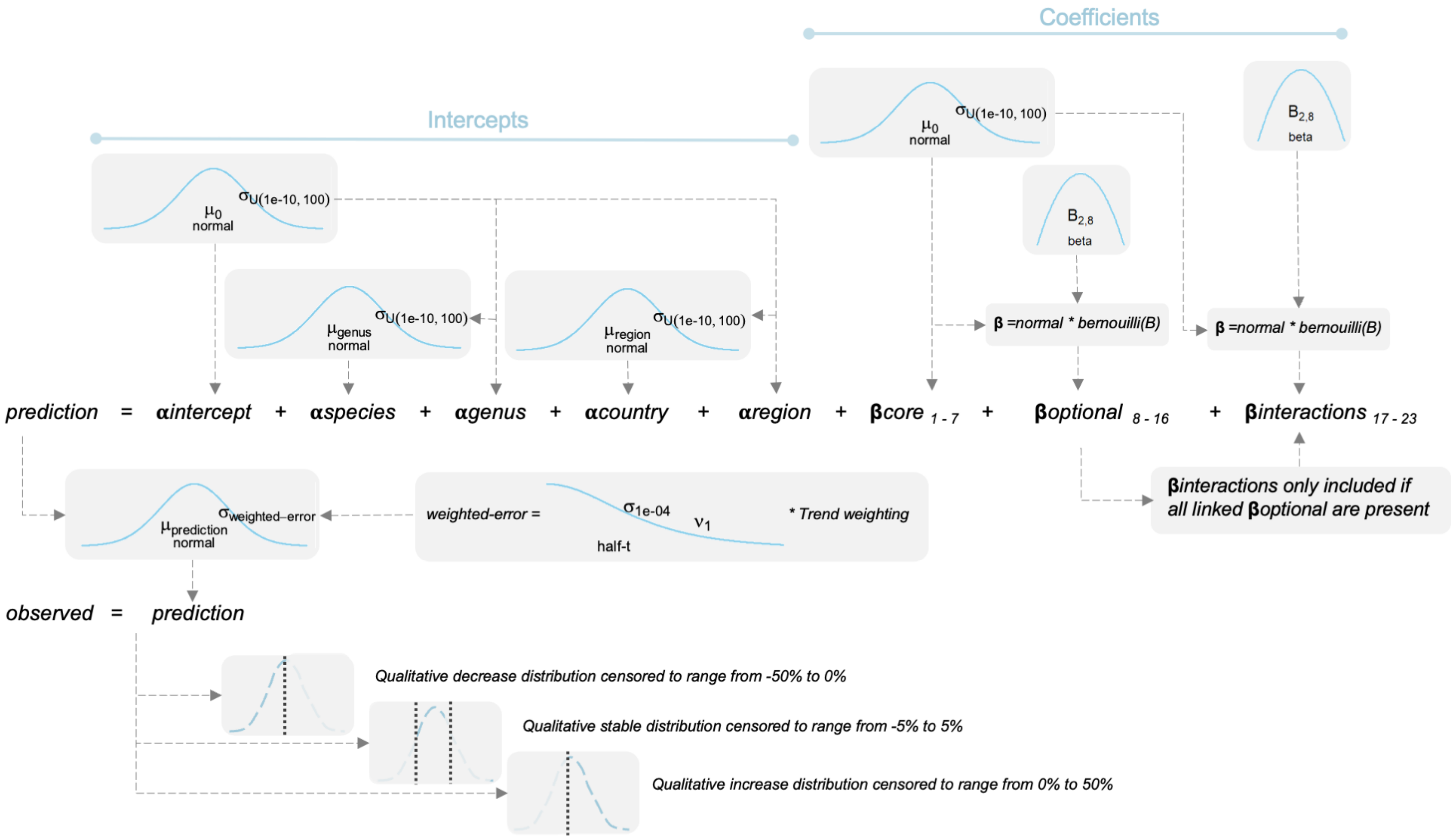

Figure S8. Model structure of hierarchical linear model, describing distributions of priors and hyperpriors, as well as the process for incorporating overall error, imputation error, trend weights (see *Weighted error* below), and censoring within the model. We use five distributions (parameters described in brackets) within the model: normal (μ = mean, σ = standard deviation), beta (shape1, shape 2), half-t distribution (μ = mean, σ = standard deviation, df = degrees of freedom), U/uniform (minimum, maximum), and Bernoulli (B = probability).

Our observed population trends fall into two types: quantitative annual rates of change and qualitative descriptions of change. Both data types were modelled with the same normal error prior (Eq2), but to deal with the different data types, and the unknown values of the qualitative descriptions, we censored the qualitative records to indicate that the true value is unknown, but it occurs within a specified range. We specified these annual rate change ranges as -50% to 0%, -5 to 5%, and 0% to 50% within the decrease, stable and increase categories, respectively.

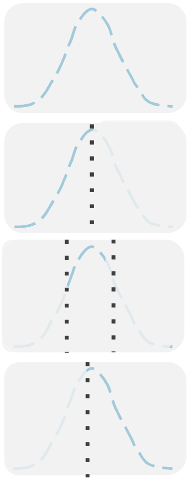

Eq. 3a. $r_o \sim$ *Uncensored distribution of quantitative records*

Eq. 3b. $r_o \sim$ *Qualitative decrease distribution censored to range from -50% to 0%*

Eq. 3c. $r_o \sim$ *Qualitative stable distribution censored to range from -5% to 5%*

Eq. 3d. $r_o \sim$ *Qualitative increase distribution censored to range from 0% to 50%*

Equation 3 is a distributional representation of Equation 2 describing how the observed annual rate of change is constrained (or censored) to occur within specific values.

The censoring range thresholds are similar to the range of the observed rates of change (-75% to 68%). Many of the qualitative records address known data biases as they occur in less-well represented regions, species, and time-periods [1]. However, these lower quality records can be more prone to error. As a result, we conduct sensitivity analysis (see Sensitivity analysis below) to assess how including censored observations altered model fit, compared to only using quantitative, and high-quality quantitative (derived from at least three abundance observations), trends.

To allow the latent rates of change to vary (***e*** in Equation 2), we set the standard deviation of the latent states normal distribution as a half-t (or half-cauchy) hyperprior (centred at zero, with a standard deviation of 0.001 and one degree of freedom). However, as some observations were likely to be more robust than others, we altered the standard deviation of the latent state normal distributions depending on each observation's quality. Specifically, we varied the standard deviation by multiplying the half-t hyperprior by a deterministic weighting term; essentially the standard deviation was inflated for lower quality observations (see *Weighted error* below).

Random intercepts

We used a hierarchical model structure to account for phylogenetic and spatial non-independence in the data, including species as a random intercept nested with genus, and country as a random intercept nested within sub-regions, as defined by the United Nations (https://www.un.org/about-us/member-states). These parameters were fit with a normal distribution centred at zero and their error terms were given a vague uniform hyper prior, with a standard deviation ranging from $1e^{-10}$ to 100.

Coefficients and covariates

With a combined 23 covariates and interactive effects, we were conscious of overparameterizing the model. As a result, we split these parameters into three groups: 1) core parameters – which included main effects that have previously been reported as influential, are expected to be influential, or control for other parameters and methodological features; 2) optional parameters – which included main effects we considered interesting but with little evidence to-date of any influence on trends; and 3) interaction parameters – which includes all interaction terms between parameters. Core parameters included: Change in human density, Primary land loss, Population area, Body mass, Change in extreme heat, Governance, and Protected area coverage. These core parameters were included in every model, but we used Kuo and Mallick variable selection [32] to identify important parameters from the optional and interaction groups, where variables were only included in an iteration if they were selected from Bernoulli priors. Our optional parameter group was assigned a Bernoulli prior, which sampled from a beta hyperprior ($a = 2, \beta = 8$), such that approximately 20% of optional effects would

be included in any iteration, on average, but this could range from 0 – 100%. The interaction parameter group had an identical, but separate prior setup. Crucially, this interaction prior was only activated if both main effect parameters were present in the model. For example, for the Change in extreme heat and Change in drought interaction to be selected, it would require Change in drought to be selected from the optional Bernoulli prior, and then the interaction itself would need to be selected from the interactive Bernoulli prior. As variable selection can be highly influenced by the standard deviation of the parameter slope coefficients, we specified the slope standard deviation as a vague uniform hyperprior ranging from $1e^{-10}$ to 100.

Imputation uncertainty

Six of the covariates in the model contained missing values that were filled using imputation (see *Land-use*, *Traits* and *Governance* within the *Covariates* section above). To improve the robustness of our model inference, we accounted for uncertainty in the imputed estimates by treating imputed values of the covariates as distributions instead of point estimates, where each imputed value was assigned a normal distribution centred at the mean imputed estimate and with an error varying by the imputed observation standard deviation. As we z-transformed all of our covariates to standardise coefficients, except 'War present' which is a categorical variable, we also had to rescale the associated imputation standard deviations. As standard deviations cannot be rescaled in the same way as the imputed estimates, we first converted the standard deviation into confidence intervals, we then z-transformed the intervals using the mean and standard deviation of the covariate, and then back calculated the standard deviation from these intervals.

Weighted error

When developing the model, we were conscious that all not rates of change should contribute equally to the fit. For example, whilst including the censored records could decrease taxonomic and spatial biases in the data, they may also introduce error, as these censored records are unlikely to be as accurate as the quantitative trends. As a result, we included a weight term to inflate the uncertainty in these lower quality records, where the half-t hyperprior discussed above is multiplied by a weight

term defined as the inverse of the estimated error in the rate of change. This weight term was developed through simulation (see below), and these simulated error weights inflated the variance around the trend in all low-quality observations, not just the qualitative ones.

When simulating the trend weights, we considered our real trend data to be estimates of true trends with some degree of error. This error would be influenced by the certainty of the population abundance estimates, the sampling intensity (e.g. Is the population sampled every year or only in 50% of years?), and the sampling duration (e.g. Is the trend based on 2 or 20 years?). As a result, we developed a simulated trend dataset comprised of 'true' trends where abundance values are known (not estimates) and complete, and an edited trend dataset where abundances are uncertain and missing as expected in a real scenario and observed in our trend dataset.

For the 'true' trend dataset, we simulated 6000 time-series of abundances which varied in duration (from 2 to 20 years), with an estimate of abundance in all years throughout that duration. We then calculated the true trend for each time-series by modelling abundances (response) against year in a log-linear regression, whilst accounting for temporal autocorrelation between abundances using the Ornstein-Uhlenbeck process. We then converted the slope estimate from this model into an annual rate of change (%). Abundance values exhibit a normal distribution ranging from approximately 0 to 500.

For the edited trend dataset, we altered two parameters in each of the 6000 time-series of abundances generated above. Firstly, for each abundance estimate in each time-series, we developed a random normal distribution, centred on the true abundance value, but with varying levels of error (coefficient of variation from 0.02 to 0.2). For example, with a true abundance of 100, a low error of 0.02 would produce a range of abundance estimates from approximately c.95 to c.105, whilst the abundance would range from c.50 to c.150 with an error of 0.2 (Figure S9). We sampled from these newly created abundance distributions to produce new error-prone abundance estimates, reminiscent of real uncertainty in abundance estimation. Secondly, we removed a random sample (between 0% and 90%)

of the observations in each time-series, producing time-series' with varying levels of completeness. We then re-calculated the annual rate of change (%) as in the true trend to produce the estimated trend. The distribution of the estimated trend was largely similar to the true trend obtained from the complete dataset (Figure S10).

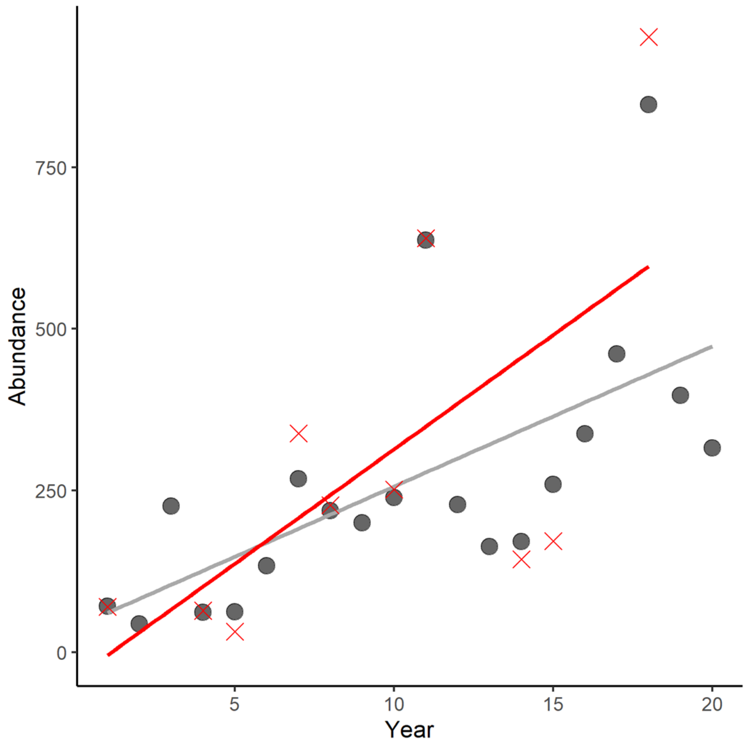

Figure S9. Impact of adding noise to abundance values and removing abundance values on the population trend, with the true trend (derived from known and complete abundance values) in grey, and the estimated ones in red. In this example, the coefficient of variation equals 0.2, with a sampling intensity of 50% i.e. half the years in the population monitoring period have abundance values.

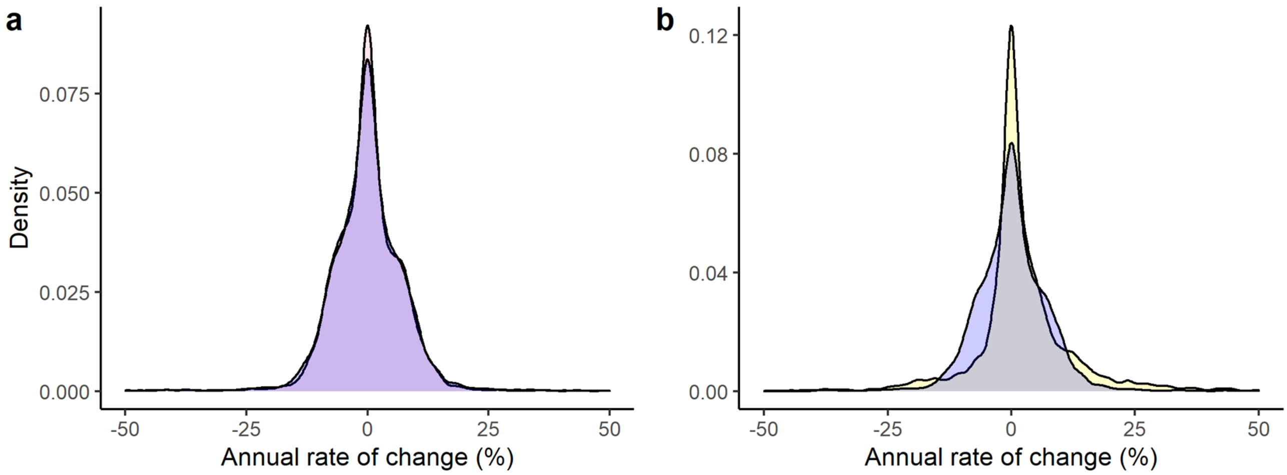

Figure S10. a) Distribution of simulated true trend values (pink) and simulated estimated trend values (blue); notably, the density plots almost perfectly overlap to produce a purple colour. b) Distribution of simulated estimated trend values (blue) and real trend values compiled from CaPTrends [33] and the Living Planet Index [2] in yellow.

We extracted the absolute error (difference) in the annual rate of change of the true and estimated trends, and modelled this error (as the response) against sampling intensity (what percentage of years have observations), the coefficient of variation around the abundance estimates, and the duration of the trend, all in a log-linear regression. Trends with a higher sampling intensity (coef = -1.59, CI: -1.72, -1.47), lower coefficient of variation (coef = 4.09, CI: 3.68, 4.49), and longer duration of the trend (coef = -0.12, CI: -0.13, -0.11), had lower errors (Figure S11). We used this model based on simulated data to predict the likely error in the real data. For sampling intensity, we calculated the percentage of abundance values used to calculate the trend relative to the trend duration. For the trend duration, we calculated the number of years in between the population monitoring period (end minus start). Unfortunately, in most cases the estimates of uncertainty around the raw abundance values were unavailable, so we were unable to directly calculate the coefficient of variation for each trend. However, we did have data describing the quality of the sampling and modelling which could act as a proxy for the accuracy of the abundance values. Specifically, we scored trends separately in three areas (Table S4), where trends could only be assigned to one category per area; we then added the score across the three areas: *Sampling* – how systematically was the population sampled? *Modelling* – how robust was the approach for modelling abundance values? *Low-quality record* – does the record meet any of the criteria for being considered low quality? For example, a trend with systematic population sampling (+0.04), in which sampling effort was accounted for (+0.04), and none of the low-quality criteria were met (+0), would be given a coefficient of variation score of 0.08. For an abundance value of 100, this coefficient of variation score would allow the abundance to vary between 75 and 125. Admittedly, our scoring criteria are arbitrary, simply designed to add uncertainty around trends that used less robust methods, rather than describe the true uncertainty in the trend. However, as these arbitrary values only contribute one feature of three in the weighting system, there impact is likely minimal, and is tested in sensitivity analysis regardless (see below).

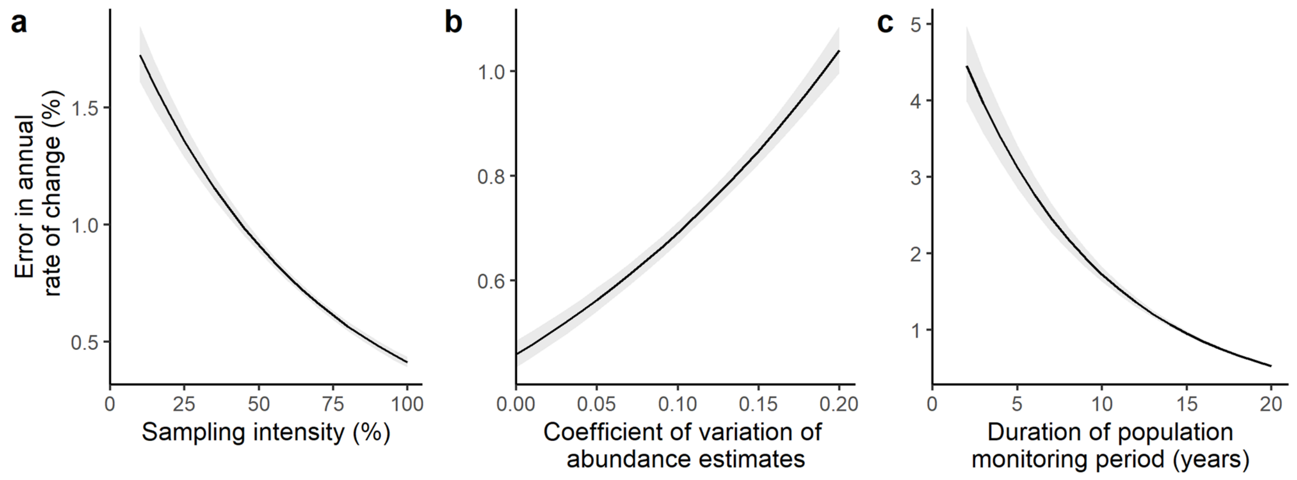

Figure S11. Marginal effect of sampling intensity (a), coefficient of variation around abundance values (b), and trend duration (c) on the absolute error in the annual rate of change (%), comparing the simulated-true to the estimated trend. Sampling intensity describes the percentage of years with abundance values in the population monitoring period. Trend duration describes the length of the population monitoring period e.g. 1990 – 1992 equals three years. Shading represents the 95% confidence intervals around the marginal effects.

Table S4. Scoring criteria used to define a coefficient of variation (CV), uncertainty, in abundances.

	**Description**	**CV**
Sampling		
	Method of population sampling is not described or is unsystematic/biased.	0.08
	Method of population sampling is systematic.	0.04
	All individuals in the population identified.	0.01
Modelling		
	Method of deriving abundance from population sampling is not described or values are just reported in their raw format.	0.08
	Sampling effort accounted for in abundance estimates.	0.04
	Abundance derived through complex modelling, or total abundance known.	0.01
Low quality record		
	Abundance values derived from genetic or harvest data; or the trend is labelled as inaccurate within the primary literature; or trend describes asymptotic instead of observed growth; or trend metric is unconventional.	0.04

After predicting the error in the real trend data using the simulated weight model, we scaled and flipped the values so that 1 indicates low error and 1e-10 indicates high error. These values had to be flipped, as the weight term in our hierarchical linear model (Figure S8) is multiplied by the precision (i.e. uncertainty) around each trend observations, in which a precision would then be deflated (i.e. uncertainty inflated) if multiplied by a high error trend. For example, for observation A with a low error of 0.9, a precision of 10 would be deflated to 6, whilst for observation B with a high error of 0.1, a precision would be deflated to 1, so A would receive 6 times more weight than B.

To ensure our weight term benefitted the model fit, we conducted sensitivity analysis to compare the model fit under four options: 1) the simulated error weight (described above); 2) weighting by trend sample size, whereby trends derived from more abundance observation are given more weight; and 3) unweighted i.e. all observations are treated equally.

Sensitivity analysis

We conducted sensitivity analysis to test how the different weighting, censoring, and temporal lag options influenced our model results, with the aim of selecting parameters which maximised model marginal and conditional R^2, whilst also balancing this decision against potential risks. For example, including censored observations may reduce model fit but this could still be worthwhile if it reduces taxonomic and spatial biases. For weighting, we ran models separately under each of the three options, including censored observations and a 5-year lag on all covariates in all cases. After identifying the simulated error weighting as the best option for maximising fit and minimising bias (see Supplementary results) we tested the censoring options, again holding all covariates at the 5-year lag. Including censored observations was valuable, so we included the censored observations when assessing the different temporal lag models, from which we identified that using a 10-year lag improved model fit. In each case, we ran the model through two chains, each with 10,000 iterations and discarding the first 5,000. We thinned the complete chains to store every other iteration (thinning factor of 2). We monitored convergence of key parameters within each model, specifically: standard deviation of the model intercept, standard deviation of beta coefficients, standard deviation of each random effect (regions, countries, genus and species), standard deviation of the overall model error, the optional parameter beta hyperprior, and the interactive parameter beta hyperprior. We ensured the multivariate potential scale reduction factor was less 1.1 across all models in the sensitivity analysis.

Model running

After selecting the simulated error weighting, censored observations, and a 10-year lag from the from the sensitivity analysis (see Sensitivity analysis in the supplementary results), we ran the full model through three chains, each with 120,000 iterations. The first 20,000 iterations in each chain were discarded, and we only stored every 10th iteration along the chain (thinning factor of 10). We opted for a large chain and burn-in due to the model complexity, and to allow a broad selection of parameter combinations to be tested under variable selection. We assessed convergence of the full model on all parameters monitored in the sensitivity analysis, as well as the model intercept, all 16 main effects and all 7 interactive effects (23 slope coefficients in total). We checked the standard assumptions of a mixed effect linear model (normal residuals and heterogeneity of variance), and tested the residuals to

ensure no spatial (Moran's test) or phylogenetic (Pagel's lambda) autocorrelation. We also conducted posterior predictive checks to ensure independently simulated values were broadly reminiscent of model predicted values.

After model running, we calculated how frequently, as a proportion, each of the 23 main and interactive effects occurred within the iterations. For the optional parameters, this was derived by dividing the frequency of occurrence by the total count of iterations. For the interactive parameters, whose inclusion was dependent on the frequency by which their derivative main effects were selected, we divided the frequency of occurrence by the total count of iterations where both derivative main effects were present. We also report the median slope coefficient and associated credible intervals for each of the main and interactive effects, and produce marginal effect plots for a selection of important parameters. These marginal effects hold all other covariates at zero (which is the equivalent of the mean, as covariates were z-transformed). We also display the distribution of the random intercepts e.g. for each region, country, genus and species.

Modelling – Counterfactual scenarios

To explore how observed changes in land-use, climate and human development have influenced population trends, we developed three counterfactual scenarios, where we compared observed population change to predicted population change if land-use, climate, and human development remained static. For instance, in the climate change counterfactual scenario, we predicted each observations population trend using the available covariate data (e.g. land-use, governance and trait covariates), as well as taxa and location data (to provide sensitivity to the models varying random intercepts), but set the climate change covariate data to zero (in this case, change in extreme heat and change in drought). Using this scenario data, we predicted each trend using the global model (all covariate parameters). We then subtracted these counterfactual predictions from the observed trends to define 'Difference in annual rate of change (%)', whereby a positive value indicates carnivore populations would be in better shape (fewer declines) under the counterfactual scenario, and vis-versa.

We summarise counterfactual scenarios by reporting the median Difference in annual rate of change and 95% quantiles across the observed 1,127 populations.

Modelling - Socio-economic development and non-linearity in carnivore trends

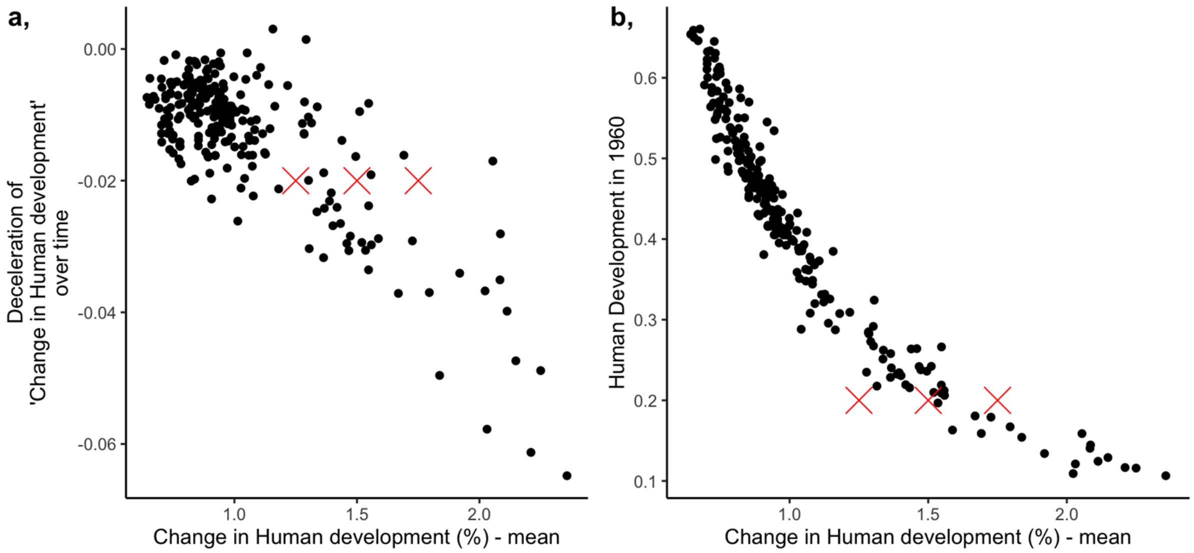

Figure S12. a) Relationship between the mean Change in human development (%) and the rate at which this change in human development decelerates in the human development data i.e. human development change decelerates faster in rapidly developing countries. Deceleration is measured as the slope coefficient of a linear model, regressing Change in human development (response variable) against year, essentially describing the mean annual decline in Change in human development over time for each country b) Mean Change in human development versus the starting (year 1960) human development value. Each point represents a country, and includes all countries, not just countries we have carnivore data for. The three red crosses, reading from left to right in each plot, represent the 'Slow', 'Moderate' and 'Fast' pathways. The impact of the deceleration and mean Change in human development can be seen in Main text: Figure 4a.

Software

All analyses were in R V4.0.3, using the following R packages: nlme_3.1-152; betareg_3.1-4; rworldmap_1.3-6; RColorBrewer_1.1-2; reshape2_1.4.4; geoR_1.8-; segmented_1.3-4; car_3.0-12; carData_3.0-5; SPEI_1.7; lmomco_2.3.7; MetricsWeighted_0.5.3; loo_2.4.1; R2jags_0.7-1; rjags_4-12; coda_0.19-4; rgeos_0.5-5; compositions_2.0-2; spdep_1.1-8; spData_0.3.10; forcats_0.5.1; stringr_1.4.0; purrr_0.3.4; readr_2.0.1; tibble_3.1.8; tidyverse_1.3.1; rvest_1.0.1; xml2_1.3.2; sf_1.0-2; ncdf4_1.18; directlabels_2021.1.13; viridis_0.6.1; viridisLite_0.4.0; ggtreeExtra_1.2.1; ggtree_3.0.4; ggnewscale_0.4.5; ggridges_0.5.3; ggpubr_0.4.0; ggeffects_1.1.1; ggstance_0.3.5;

ggplot2_3.3.6; norm_1.0-9.5; phytools_1.0-1; maps_3.4.0; Rphylopars_0.3.2; ape_5.6-1; lme4_1.1-28; Matrix_1.3-4; lattice_0.20-44; mice_3.14.0; data.table_1.14.0; reshape_0.8.8; tidyr_1.2.0; dplyr_1.0.10; plyr_1.8.6; rgdal_1.5-23; raster_3.4-13; sp_1.4-6

Supplementary Results

Sensitivity analysis

We assessed how trend weighting, including censored observations, and specifying a lag period on the covariates influenced model fit and inference, in part to assess if results were particularly sensitive to specific parameters, but also to help choose the parameters which optimised fit and spatio-taxonomic coverage. Using censored observations and a lag period of 5 years on covariates, model fit was greater when using the simulated error weights compared to the unweighted model and the model weighted by sample size (Table S5). Using simulated error weights and a lag period of 5 years on covariates, we then tested the impact of including censored observations which showed higher marginal and conditional R^2 when censored observations were included. Using only high quality time-series (compared to including censored observation) resulted in a higher conditional R^2, but at the cost of excluding 19 countries and 2 species from the dataset. We considered the gain in model fit did not outweigh the added spatial and taxonomic coverage. Finally, using simulated error weights and the full dataset (including censored observations), we tested how the lag period of covariates influenced fit. All lag periods offered a similar fit (marginal and conditional R^2), and so we selected the lag most supported by the literature – 10 years, with suggestions peak population change occurs 8 years after environmental change (specifically forest loss) in mammals [27].

Table S5. Fit of nine models tested in the sensitivity analyses split across three categories: Weighting – influence of different trend observation weighting options; Censoring – impact of including different qualities of trend data; and Lag – fit under different lag periods for covariates (e.g. for a predator population monitored between 1995-2000, the Change in human density would be measured from 1995-2000, 1990-2000, and 1985-2000, respectively under the 0, 5, and 10-year lags. Fit measured as the marginal and conditional R^2. There are varying levels of data in each model, and we summarise the frequency of countries and species this data occurs in. For the weighting models, all quantitative and qualitative-censored trends were included, with a 5-year lag on the covariates. For the censoring models, all trend observations were weighted by the simulated error, with a 5-year lag on the covariates. For the lag model, all quantitative and qualitative-censored trends were included and weighted by the simulated error.

	Category	Marg. R^2	Cond. R^2	Countries (N)	Species (N)
**Weighting**	Unweighted	0.09	0.23	75	50
	Weighted by trend sample size	0.10	0.27	75	50
	Weighted by simulated error	0.11	0.27	75	50
**Censoring**	High quality time-series trends	0.11	0.28	56	48
	All quantitative trends	0.10	0.27	69	49
	All quantitative and qualitative-censored trends	0.11	0.27	75	50
**Lag**	0 years	0.10	0.28	75	50
	5 years	0.11	0.27	75	50
	10 years	0.11	0.27	75	50

In our final model, we used the simulated error weights, included censored observations, and used a 10-year covariate lag. While these decisions optimized fit and data coverage, the type of weightings, the data or time lags used had little impact on inference, as model coefficients were largely similar across all parameter types (Figure S13 – S16).

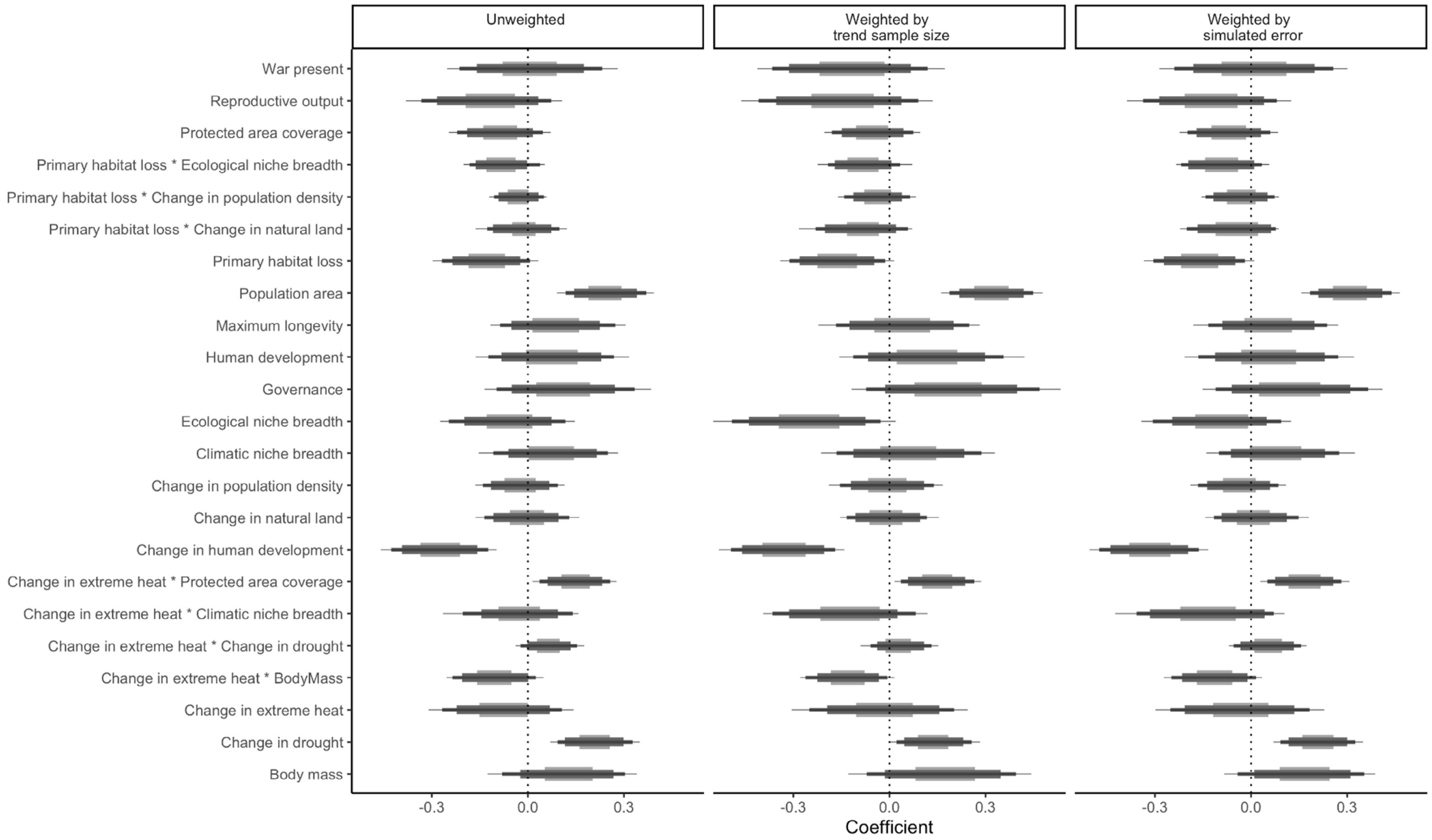

Figure S13. Standardised slope coefficients for the 23 main effects and interactions on the annual rate of change, comparing three models with different types of trend weighting: 1) trend values are unweighted; 2) trend values are weighted by the sample size (frequency of abundance observations used to derive trend); and 3) trend values are weighted by the simulated error. The four widths of the error bars represent different credible intervals: 50% (thickest), 80%, 95%, and 97.5% (thinnest)

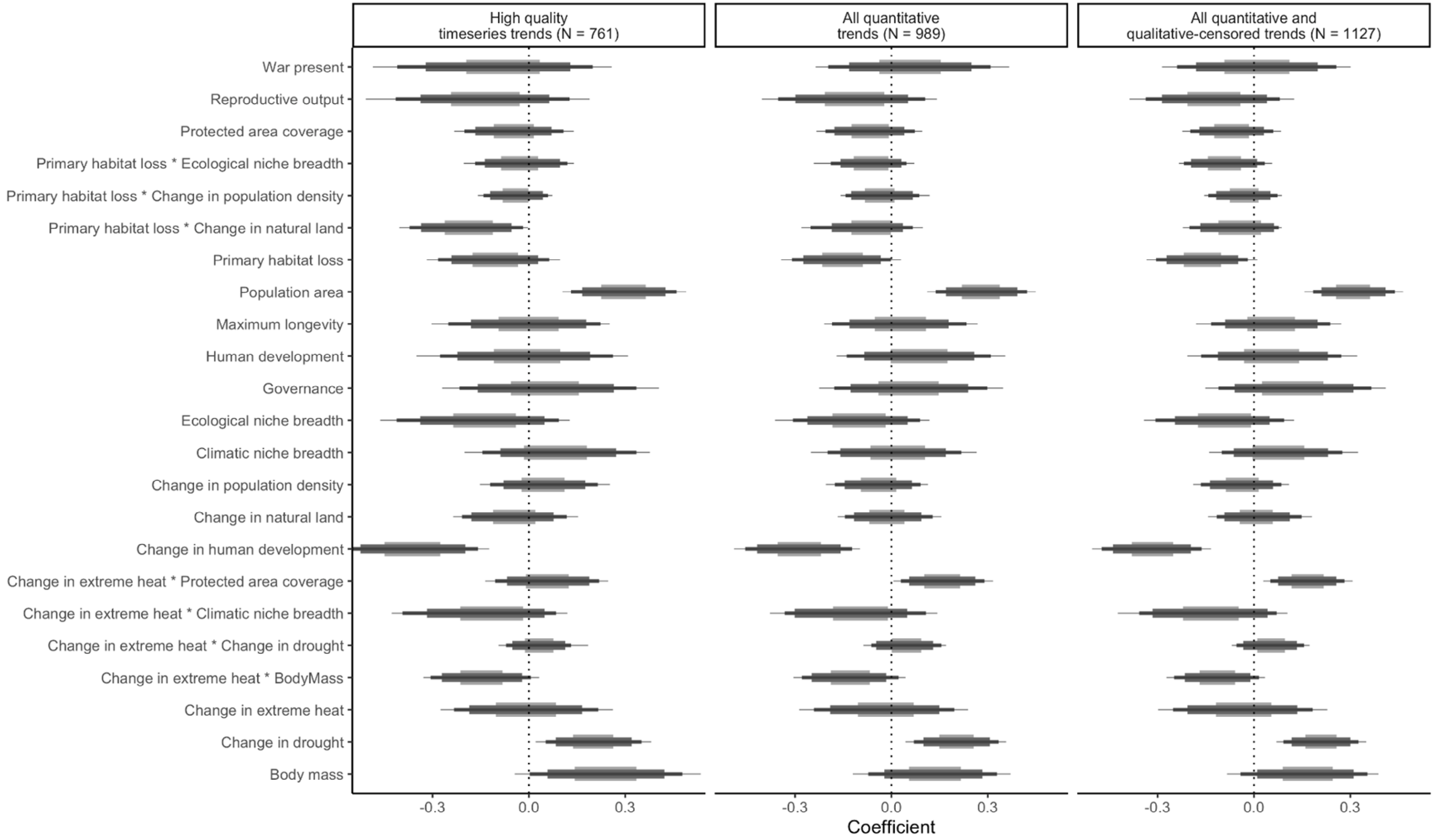

Figure S14. Standardised slope coefficients for the 23 main effects and interactions on the annual rate of change, comparing three models with different levels of inclusion for the trend data: 1) all time-series trends with at least three abundance values are used; 2) all quantitative trend values are used; and 3) all trend values are used. The numbers in brackets alongside the facet titles describe the sample size of trends in the model. The four widths of the error bars represent different credible intervals: 50% (thickest), 80%, 95%, and 97.5% (thinnest)

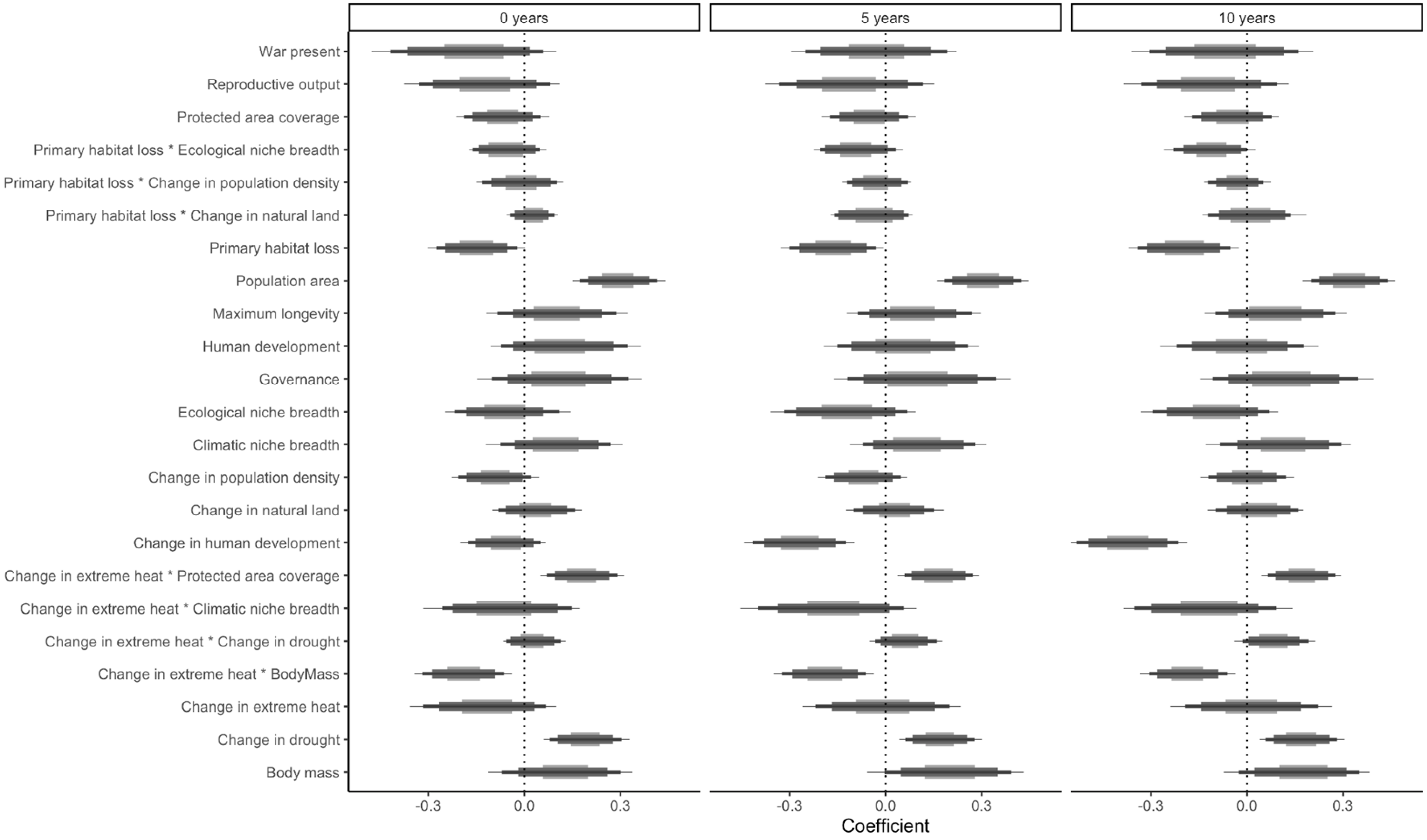

Figure S15. Standardised slope coefficients for the 23 main effects and interactions on the annual rate of change, comparing three models describing different lags of the model covariates: 1) 0 years; 2) 5 years; and 3) 10 years. The different lag periods only effect covariates that measure a change in the covariate over time. For example, for a predator population monitored between 1995-2000, the Change in human density would be measured from 1995-2000, 1990-2000, and 1985-2000, respectively under the 0, 5, and 10-year lags. The four widths of the error bars represent different credible intervals: 50% (thickest), 80%, 95%, and 97.5% (thinnest).

Parameter frequency and effects

Under variable selection within our model, some parameters occurred far more frequently than others (Figure S16). Generally, parameters with an effect at the 95% credible intervals were likely to occur more frequently, which is a consequence of the model identifying and favouring the inclusion of important variables. Change in human development was the most common optional parameter, occurring in all of the selected iterations. In contrast, Change in extreme heat * Protected area coverage was the most common interaction parameter (Figure S16).

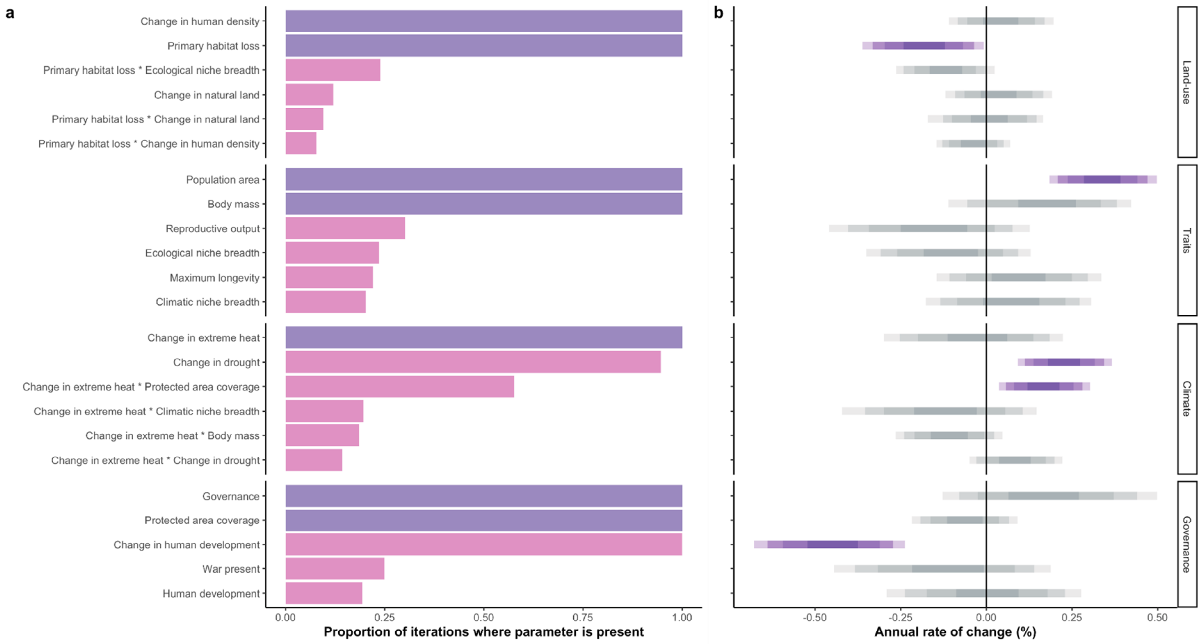

Figure S16. a) Proportion of iterations where parameter is present. Core parameters (purple) are present in all models, whilst optional and interaction parameters (pink) only occur in models when they are selected from Bernoulli distributions. b) Standardised coefficients of main effect and interactive model parameters. Parameters in purple have an effect at the 95% credible interval, whilst those in grey do not. The different credible interval thresholds are shown for each parameter, with darkest centre representing the 50% credible intervals, followed by the 80%, 90%, and 95% thresholds (the maximum and minimum point on each bar).

Model assumptions and checks

The inference model passed all standard linear mixed effect model assumptions, with residuals not showing signal of spatial or phylogenetic autocorrelation (Figure S17). However, the inference model failed to represent the more extreme observed annual rate of change (%) values, with the predicted rates of change largely ranging from -2 to 2, whilst the observed rates of change range -5 to 5; both on

an inverse hyperbolic sine scale (Figure S18a). The qualitative-censored predictions largely agreed with the observed data, where censored-increasing values were primarily predicted to increase, and censored-stable values exhibited small increases and decreases (Figure S18b). The only category with reasonably poor alignment was censored-decreasing, where populations were predicted to be both increasing and decreasing. Our posterior predictive checks suggest the model produced broadly plausible values, with the independently simulated values occurring within the distribution of the observed trends, albeit failing to represent extreme values (Figure S18c). For the qualitative-censored trends, the quasi-observed values matched the simulated values almost identically (Figure S18d), which is to be expected. The inference model had a median root mean square error of 9.2%, a median marginal R^2 of 0.15, and a median conditional R^2 of 0.4 (Figure S19).

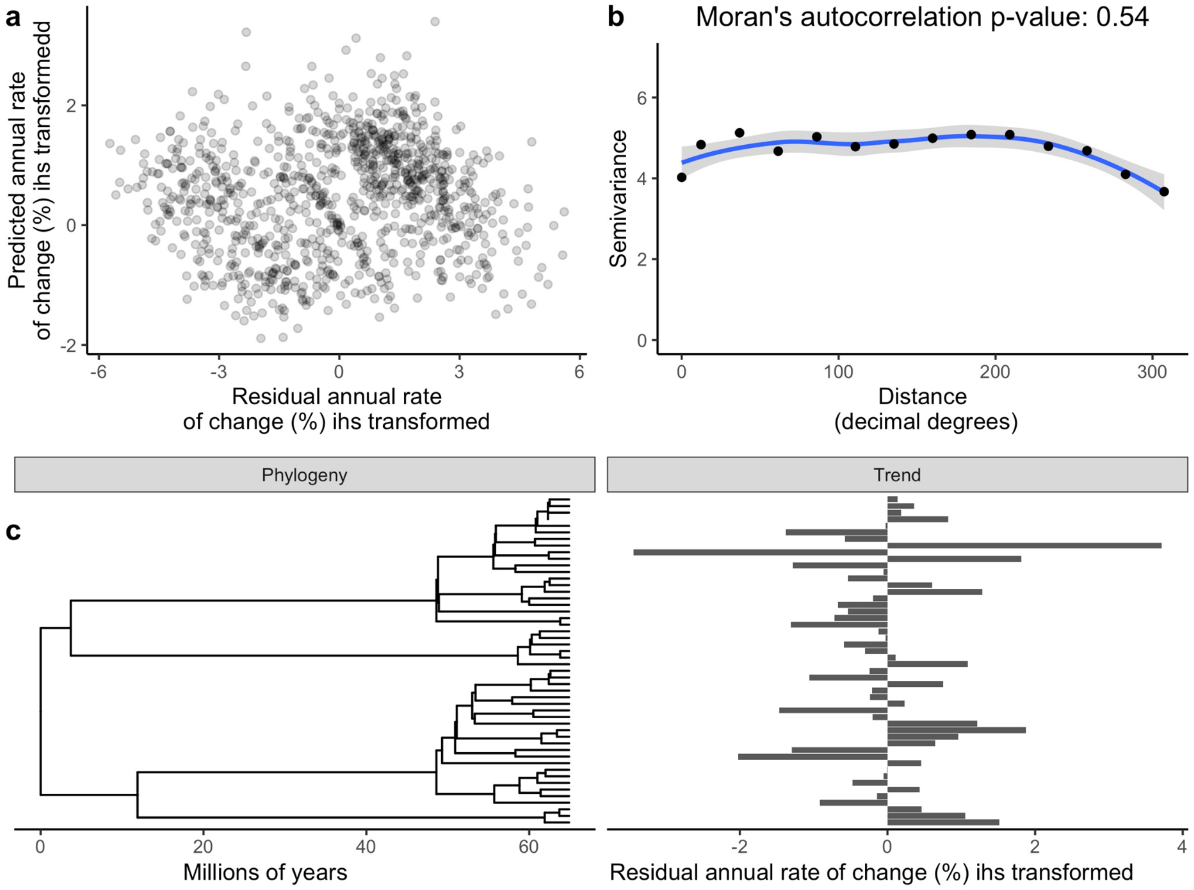

Figure S17. a) Median predicted annual rate of change (%) values from the inference model plotted against the median residual rates of change (%), both displayed with an inverse hyperbolic sine (ihs) transformation – the transformation used on the annual rate of change (%) within the inference model. b) Semivariance and Moran's autocorrelation (2-sided) of inference model's median residual annual rate of change (%) across distance/space

(decimal degrees), with 95% confidence intervals. c) The median residual annual rate of change (%) averaged (mean) across each species, plotted on the species' phylogeny; with no evidence of phylogenetic autocorrelation (Pagel's lambda = 0, p = 1). The annual rate of change (%) is ihs transformed.

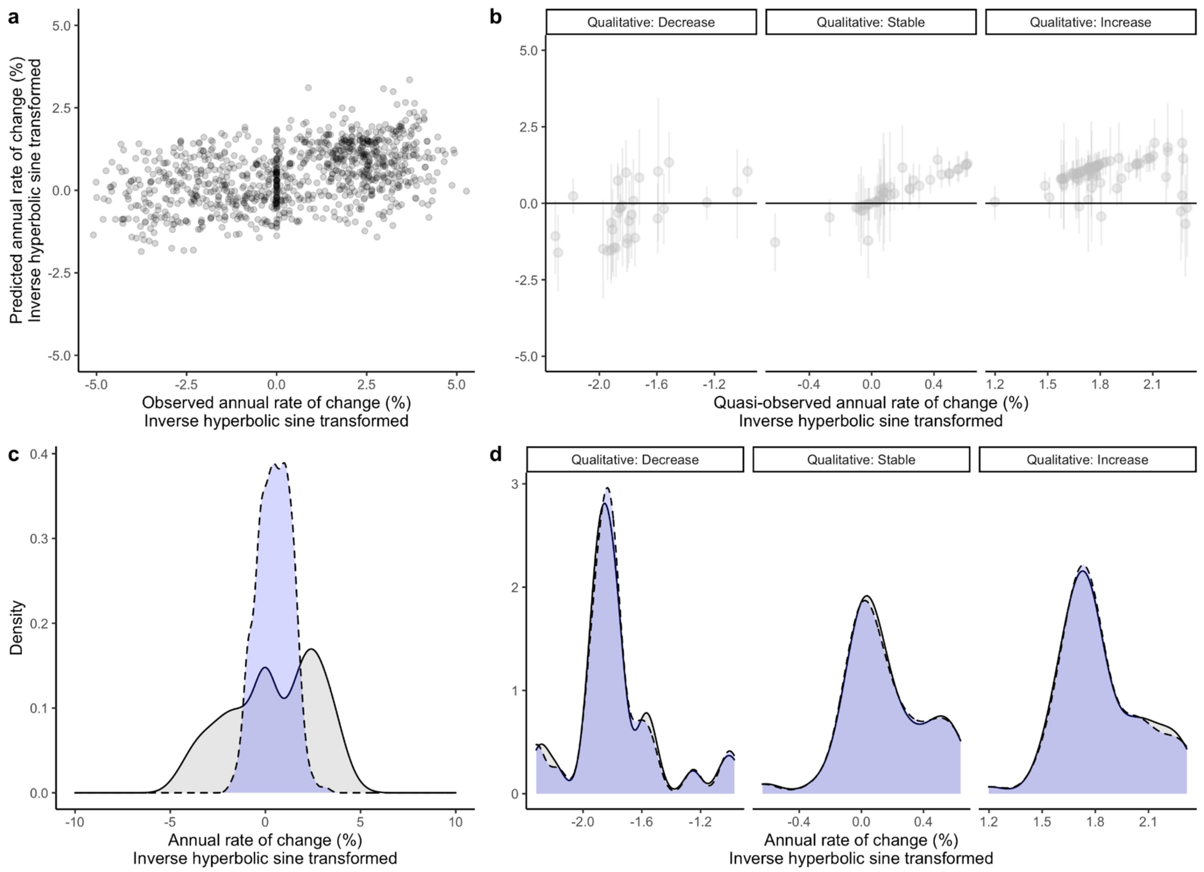

Figure S18. a) Median predicted annual rate of change (%) values from the inference model plotted against the observed rates of change (%), both displayed with an inverse hyperbolic sine (ihs) transformation – the transformation used on the annual rate of change (%) within the inference model. b) Median predicted annual rate of change (%) values (and 95% credible intervals) from the inference model plotted against each category of qualitative-censored values (median quasi-observed rates of change) - both displayed with an inverse hyperbolic sine (ihs) transformation. Values are quasi-observed as the true observed values are unknown. c) Distribution of observed annual rates of change (grey), compared to model simulated median annual rates of change (blue). d) Distribution of median quasi-observed annual rates of change (grey), compared to model simulated annual rates of change (blue).

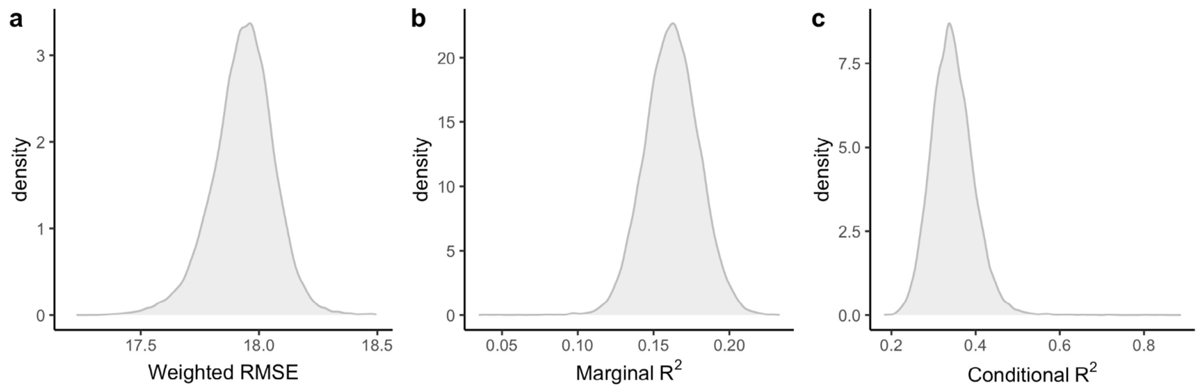

Figure S19. a) Distribution of the weighted root-mean-square error of the annual rate of change (%) in the inference model, comparing true to predicted values. b) Distribution of the inference model's marginal R^2. c) Distribution of the inference model's conditional R^2.

<u>Spatial and temporal biases in the predictors</u>

As our data exhibit spatial and temporal biases, there is a risk that our predictors don't fairly represent past climate, land-use and governance changes. For instance, if population trends only occur in countries with high governance, our ability to assess features like the impact of human development change on population trends will be limited, as we will not capture the full predictor parameter space. To assess if we are capturing the parameter space for our most influential predictor (human development change), we compare the observed values in our models to a random sample (N = 1000) at each of the following scales: global, in the United States, and in Tanzania – working between the time frame of 1960 – 2015 in each. At the global scale, this involved randomly selecting a country and a year between 1960 and 2015, we then took data from the five years either side of this year and derived the annual rate of change (%) by fitting a log-linear model of human development against year. At the national scale, we skipped the country selection stage and extracted human development change solely from the relevant country at different randomly selected time points. We repeated this process 1000 times (Figure S20).

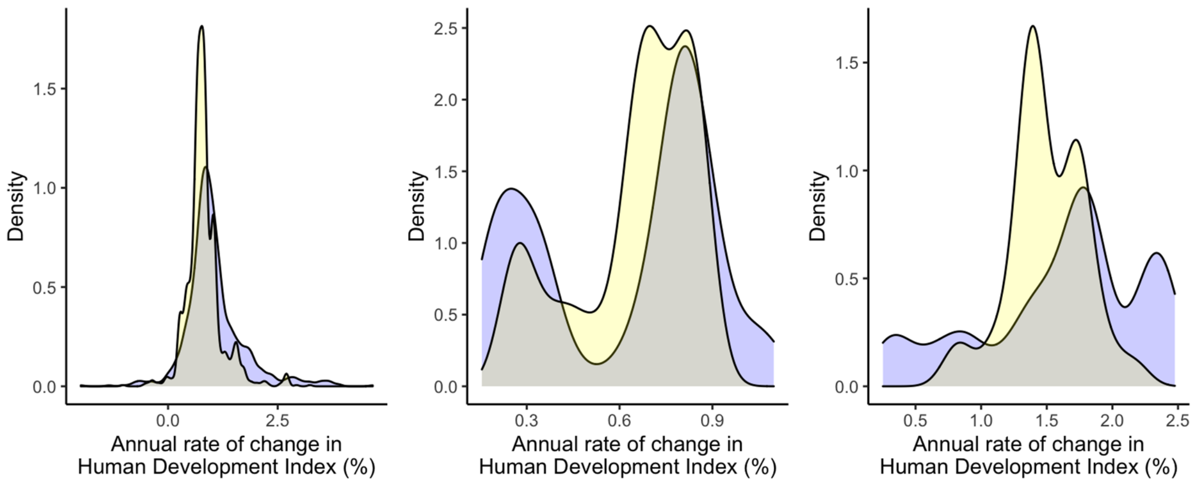

Figure S20. Distribution of change in human development (%), at the global scale (left), in the United States – the country with the most data (centre), and Tanzania – a country with lots of data but low human development values (right). Yellow shading is the observed data used in our models, whilst the blue shading represents a random sample of 1000 rate of changes in human development. At the global scale (left), this involved randomly selecting a country and a year between 1960 and 2015, we then took data from the five years either side of this year and derived the annual rate of change (%) by fitting a log-linear model of human development against year. In the national plots (centre and right), we skipped the country selection stage and extracted values solely from the relevant country. We repeated this process 1000 times.

This analysis shows that our observed human development change data closely matches the random sample at both the global and national scales. This analysis suggests, that whilst spatial and temporal biases are present, we are at least capturing much of the parameter space of our most influential variable

Quadratic modelling of human development

As earlier work has found that human development has a quadratic effect on wildlife population trends [21] - a feature we do not test in our model presented in Figure S8 - here, using the quantitative records (N = 983) and a simplified model, we test if our significant human development change result persists when we allow the human development score to modelled with a quadratic term. Specifically, we regressed annual rates of change (%; inverse hyperbolic-sine transformed) against human development change and a second-order polynomial of human development (i.e. quadratic), with a random intercept of species nested in genera and countries within UN sub-regions. This simplified model supported the main conclusions from the manuscript, where human development change

remains a more important factor than human development score, even after accounting for a quadratic relationship between rate of change and human development score (Table S6).

Table S6. Fixed effects from our simplified quadratic human development model

**Term**	**95% Confidence intervals**
Human development change	-1.33, -0.32
Human development (polynomial 1)	-7.34, 14.75
Human development (polynomial 2)	-7.47, 6.55

Supplementary References

1. Johnson, T. F., Cruz, P., Isaac, N. J. B., Paviolo, A. & González-Suárez, M. CaPTrends: A database of large carnivoran population trends from around the world. *Glob. Ecol. Biogeogr.* **n/a**, (2022).
2. WWF. Living Planet Index: Data Portal. *Living Planet Index* (2020).
3. Hurtt, G. C. *et al.* Harmonization of global land use change and management for the period 850-2100 (LUH2) for CMIP6. *Geosci. Model Dev.* (2020) doi:10.5194/gmd-13-5425-2020.
4. Florczyk, A. J. *et al. GHSL Data Package 2019*. https://ghsl.jrc.ec.europa.eu/download.php?ds=pop (2019).
5. CHELSAcruts - High resolution temperature and precipitation timeseries for the 20th century and beyond - EnviDat. https://www.envidat.ch/dataset/chelsacruts.
6. Karger, D. N. *et al.* Climatologies at high resolution for the earth's land surface areas. *Sci. Data* **4**, 170122 (2017).
7. Pettersson, T. *UCDP/PRIO Armed Conflict Dataset Codebook Version 19.1*. (2019).
8. Kaufmann, D., Kraay, A. & Mastruzzi, M. The worldwide governance indicators: Methodology and analytical issues. *Hague J. Rule Law* (2011) doi:10.1017/S1876404511200046.
9. UNDP. Human Development Index (HDI). (2021).

10. UNEP-WCMC & IUCN. Protected Planet: The World Database on Protected Areas (WDPA). *Available Wwwprotectedplanetnet* (2021).
11. IUCN. Spatial Data Download. (2020).
12. IUCN. Habitats Classification Scheme (Version 3.1). (2020).
13. Fick, S. E. & Hijmans, R. J. WorldClim 2: new 1-km spatial resolution climate surfaces for global land areas. *Int. J. Climatol.* (2017) doi:10.1002/joc.5086.
14. Jones, K. E. *et al.* PanTHERIA: a species-level database of life history, ecology, and geography of extant and recently extinct mammals. *Ecology* **90**, 2648–2648 (2009).
15. De MagalhÃes, J. P. & Costa, J. A database of vertebrate longevity records and their relation to other life-history traits. *J. Evol. Biol.* (2009) doi:10.1111/j.1420-9101.2009.01783.x.
16. Spooner, F. E. B., Pearson, R. G. & Freeman, R. Rapid warming is associated with population decline among terrestrial birds and mammals globally. *Glob. Change Biol.* (2018) doi:10.1111/gcb.14361.
17. Thornthwaite, C. W. An Approach toward a Rational Classification of Climate. *Geogr. Rev.* (1948) doi:10.2307/210739.
18. Vicente Serrano, S. M., Beguiria, S. & Lopez-Moreno, J. I. A multi-scalar drought index sensitive to global warming: the standardised precipitation evapotranspiration index. *J. Clim.* (2010).
19. Braga-Pereira, F., Peres, C. A., Campos-Silva, J. V., Santos, C. V.-D. & Alves, R. R. N. Warfare-induced mammal population declines in Southwestern Africa are mediated by species life history, habitat type and hunter preferences. *Sci. Rep.* **10**, 15428 (2020).
20. Amano, T. *et al.* Successful conservation of global waterbird populations depends on effective governance. *Nature* **553**, 199–202 (2018).
21. Barnes, M. D. *et al.* Wildlife population trends in protected areas predicted by national socio-economic metrics and body size. *Nat. Commun.* **7**, 12747 (2016).
22. Van Buuren, S. & Groothuis-Oudshoorn, K. MICE: Multivariate Imputation by Chained Equations in R. *J. Stat. Softw.* **10**, 1–68 (2011).

23. Prados de la Escosura, L. Augmented human development in the age of globalization†. *Econ. Hist. Rev.* **74**, 946–975 (2021).

24. González-Suárez, M. & Revilla, E. *Carnivora trait dataset*. (2014).

25. Goolsby, E. W., Bruggeman, J. & Ané, C. Rphylopars : fast multivariate phylogenetic comparative methods for missing data and within-species variation. *Methods Ecol. Evol.* **8**, 22–27 (2017).

26. Johnson, T. F., Isaac, N. J. B., Paviolo, A. & González-Suárez, M. Handling missing values in trait data. *Glob. Ecol. Biogeogr.* **30**, (2021).

27. Nyakatura, K. & Bininda-Emonds, O. R. P. Updating the evolutionary history of Carnivora (Mammalia): A new species-level supertree complete with divergence time estimates. *BMC Biol.* (2012) doi:10.1186/1741-7007-10-12.

28. Daskalova, G. N. *et al.* Landscape-scale forest loss as a catalyst of population and biodiversity change. *Science* (2020) doi:10.1126/science.aba1289.

29. Plummer, M. rjags: Bayesian graphical models using MCMC. *R Package Version 3-13* (2016).

30. R Development Core Team. R Development Core Team, R: a language and environment for statistical computing. *R Lang. Environ. Estat. Comput.* (2020).

31. Burbidge, J. B., Magee, L. & Robb, A. L. Alternative Transformations to Handle Extreme Values of the Dependent Variable. *J. Am. Stat. Assoc.* **83**, 123–127 (1988).

32. Kuo, L. & Mallick, B. Variable selection for regression models. *Sankhyā Indian J. Stat. Ser. B* (1998).

33. Johnson, T. F., Cruz, Paula., Isaac, N. J. B., Paviolo, A. & Gonzalez-Suarez, M. CaPTrends: A global database of population trends in large terrestrial Carnivorans. *Unpublished* (2021).
