# Supplementary material for: Socioeconomic factors predict population changes of large carnivores better than climate change or habitat loss
Source: Nat Commun. 2023 Jan 24;14:74. doi: 10.1038/s41467-022-35665-9 (PMC9873912; doi:10.1038/s41467-022-35665-9)
Supplement: Supplementary file 2 — Reporting Summary [file 41467_2022_35665_MOESM2_ESM.pdf]

nature portfolio

Corresponding author(s): Thomas Frederick Johnson

Last updated by author(s): 24/11/2022

Reporting Summary

Nature Portfolio wishes to improve the reproducibility of the work that we publish. This form provides structure for consistency and transparency in reporting. For further information on Nature Portfolio policies, see our Editorial Policies and the Editorial Policy Checklist.

Statistics

For all statistical analyses, confirm that the following items are present in the figure legend, table legend, main text, or Methods section.

n/a	Confirmed	
☐	☒	The exact sample size (*n*) for each experimental group/condition, given as a discrete number and unit of measurement
☐	☒	A statement on whether measurements were taken from distinct samples or whether the same sample was measured repeatedly
☐	☒	The statistical test(s) used AND whether they are one- or two-sided<br>*Only common tests should be described solely by name; describe more complex techniques in the Methods section.*
☐	☒	A description of all covariates tested
☐	☒	A description of any assumptions or corrections, such as tests of normality and adjustment for multiple comparisons
☐	☒	A full description of the statistical parameters including central tendency (e.g. means) or other basic estimates (e.g. regression coefficient) AND variation (e.g. standard deviation) or associated estimates of uncertainty (e.g. confidence intervals)
☐	☒	For null hypothesis testing, the test statistic (e.g. *F*, *t*, *r*) with confidence intervals, effect sizes, degrees of freedom and *P* value noted<br>*Give P values as exact values whenever suitable.*
☐	☒	For Bayesian analysis, information on the choice of priors and Markov chain Monte Carlo settings
☐	☒	For hierarchical and complex designs, identification of the appropriate level for tests and full reporting of outcomes
☐	☒	Estimates of effect sizes (e.g. Cohen's *d*, Pearson's *r*), indicating how they were calculated

Our web collection on statistics for biologists contains articles on many of the points above.

Software and code

Policy information about availability of computer code

Data collection	We used Google chrome to download datasets.
Data analysis	We used R V4.0.3 for all analysis. Our code is available at the following link with a CC-BY license upon acceptance:<br><br>We used the following R packages: nlme_3.1-152; betareg_3.1-4; rworldmap_1.3-6; RColorBrewer_1.1-2; reshape2_1.4.4; geoR_1.8-; segmented_1.3-4; car_3.0-12; carData_3.0-5; SPEI_1.7; lmomco_2.3.7; MetricsWeighted_0.5.3; loo_2.4.1; R2jags_0.7-1; rjags_4-12; coda_0.19-4; rgeos_0.5-5; compositions_2.0-2; spdep_1.1-8; spData_0.3.10; forcats_0.5.1; stringr_1.4.0; purrr_0.3.4; readr_2.0.1; tibble_3.1.8; tidyverse_1.3.1; rvest_1.0.1; xml2_1.3.2; sf_1.0-2; ncdf4_1.18; directlabels_2021.1.13; viridis_0.6.1; viridisLite_0.4.0; ggtreeExtra_1.2.1; ggtree_3.0.4; ggnewscale_0.4.5; ggridges_0.5.3; ggpubr_0.4.0; ggeffects_1.1.1; ggstance_0.3.5; ggplot2_3.3.6; norm_1.0-9.5; phytools_1.0-1; maps_3.4.0; Rphylopars_0.3.2; ape_5.6-1; lme4_1.1-28; Matrix_1.3-4; lattice_0.20-44; mice_3.14.0; data.table_1.14.0; reshape_0.8.8; tidyr_1.2.0; dplyr_1.0.10; plyr_1.8.6; rgdal_1.5-23; raster_3.4-13; sp_1.4-6

For manuscripts utilizing custom algorithms or software that are central to the research but not yet described in published literature, software must be made available to editors and reviewers. We strongly encourage code deposition in a community repository (e.g. GitHub). See the Nature Portfolio guidelines for submitting code & software for further information.

Data

Policy information about availability of data

All manuscripts must include a data availability statement. This statement should provide the following information, where applicable:

- Accession codes, unique identifiers, or web links for publicly available datasets
- A description of any restrictions on data availability
- For clinical datasets or third party data, please ensure that the statement adheres to our policy

All data is openly accessible subject to licence conditions. Trend data was sourced from CaPTrends (https://onlinelibrary.wiley.com/doi/full/10.1111/geb.13587) and the Living Planet (https://www.livingplanetindex.org/data_portal). Covariate data: climate (https://chelsa-climate.org/), land-use (https://luh.umd.edu/), governance (https://databank.worldbank.org/source/worldwide-governance-indicators), human development (https://hdr.undp.org/data-center/human-development-index), PanTHERIA traits (https://esajournals.onlinelibrary.wiley.com/doi/10.1890/08-1494.1), AnAge traits (https://genomics.senescence.info/species/index.html), and protected areas (https://www.protectedplanet.net/en/thematic-areas/wdpa). All data is described in Supplementary Table 2, with extended descriptions of how to access and use data within the code (https://zenodo.org/badge/latestdoi/555432324).

Field-specific reporting

Please select the one below that is the best fit for your research. If you are not sure, read the appropriate sections before making your selection.

☐ Life sciences ☐ Behavioural & social sciences ☒ Ecological, evolutionary & environmental sciences

For a reference copy of the document with all sections, see nature.com/documents/nr-reporting-summary-flat.pdf

Ecological, evolutionary & environmental sciences study design

All studies must disclose on these points even when the disclosure is negative.

Study description	In this study, we assess how rates of change in carnivore population abundances are influenced by climate, land-use, governance and trait characteristics. We test for an effect in 16 covariates (and a further 7 interactions) using a hierarchal linear model. This model includes many novel features, including our approach for handling measurement error in both the response and predictors, and further novelty by using a data integration to combine quantitative and qualitative data in one modeling framework.
Research sample	Our sample describes the population trends of carnivores (order Carnivora) from the families Canidae, Felidae, Hyaenidae, and Ursidae - some of the most charismatic and functionally important fauna on the planet. Out of the 87 species in these families, we obtained trend information for 50. Trend information came primarily from CapTrends (see above), and a smaller sample came from the Lining Planet data. This trend data is biased towards larger and more charismatic speices. For instance, nearly 50% of trend records come from just 10 species. Our research sample describes unique abundance trend observations (N = 1123). We have used all available data and no power analysis was used to determine sample size.
Sampling strategy	We did not conduct power analysis to pre-determine sample sizes. Sample size was maximized by using all available data on carnivore trends. Our sample size of 1123 trends offers approximately 50 trend observation per covariate parameter.
Data collection	In this study, we compiled data collection from an array of internet sources. Trend data was sourced from CaPTrends (https://onlinelibrary.wiley.com/doi/full/10.1111/geb.13587) and the Living Planet (https://www.livingplanetindex.org/data_portal). Covariate data: climate (https://chelsa-climate.org/), land-use (https://luh.umd.edu/), governance (https://databank.worldbank.org/source/worldwide-governance-indicators), human development (https://hdr.undp.org/data-center/human-development-index), PanTHERIA traits (https://esajournals.onlinelibrary.wiley.com/doi/10.1890/08-1494.1), AnAge traits (https://genomics.senescence.info/species/index.html), and protected areas (https://www.protectedplanet.net/en/thematic-areas/wdpa). All data is described in Supplementary Table 2, with extended descriptions of how to access and use data within the code (https://zenodo.org/badge/latestdoi/555432324).
Timing and spatial scale	We have population trend data extending from January 1970 to December 2015, but with strong temporal bias, were 80% of trends occur after 1995, and 94% occur after 1990. Trends occur from sites around the world, but with strong spatial biases (see Figure S2), where essentially, the only locations we have more trends than species are North America, Europe, India, Indo-malaya, East Africa and South Africa. Our temporal extent was limited to after 1970 as this is conventional across biodiversity change studies.
Data exclusions	We opted to remove a selection of the population trend and covariate data as the values were deemed unreliable or unsuitable. Specifically, we removed any population trend records beginning before 1970 or after 2016 (N = 11), where governance data was largely incomplete. We also removed records overlapping multiple countries (N = 10), and any population trends with an excessively large population buffer-area (N = 40) – we set the threshold at 2 million km2 which could accommodate state and small-country level estimates, but would exclude large countries. For example, the largest population area in the dataset covered all of Russia (~21 million km2). Any population trends discussing non-native species were removed (N = 6), as well as records not overlapping any land (N = 4) e.g. Ursus maritimus populations occurring exclusively on sea-ice. We also removed any population trends where the population had either recolonised an area or become locally extinct (N = 80), which represent an extreme form of population change that could skew our inference. For instance, the drivers of local-scale rapid extinction could be very different (e.g. disease) to normal populaton drivers. After excluding records, we were left with 985 estimates of annual rate of change, and 138 qualitative descriptions of change.
Reproducibility	All code and data will be available to permit scrutiny of the work. We also include a sample of the code and data that can be run to

support future use-cases.

Randomization	N/A. All covariates fall along a spectrum of continuous values
Blinding	Blinding was not relevant to the observational nature of our work.

Did the study involve field work? ☐ Yes ☒ No

Reporting for specific materials, systems and methods

We require information from authors about some types of materials, experimental systems and methods used in many studies. Here, indicate whether each material, system or method listed is relevant to your study. If you are not sure if a list item applies to your research, read the appropriate section before selecting a response.

Materials & experimental systems

n/a	Involved in the study
☒	☐ Antibodies
☒	☐ Eukaryotic cell lines
☒	☐ Palaeontology and archaeology
☒	☐ Animals and other organisms
☒	☐ Human research participants
☒	☐ Clinical data
☒	☐ Dual use research of concern

Methods

n/a	Involved in the study
☒	☐ ChIP-seq
☒	☐ Flow cytometry
☒	☐ MRI-based neuroimaging
